# Casting votes of antecedents play a key role in successful sequential decision-making

**DOI:** 10.1371/journal.pone.0282062

**Published:** 2023-02-24

**Authors:** Mariko I. Ito, Akira Sasaki

**Affiliations:** 1 Institute of Industrial Science, The University of Tokyo, Meguro-ku, Tokyo, Japan; 2 Research Center for Integrative Evolutionary Science, The Graduate University for Advanced Studies, SOKENDAI, Hayama, Kanagawa, Japan; 3 Evolution and Ecology Program, International Institute for Applied Systems Analysis, Laxenburg, Austria; International Institute of Information Technology, INDIA

## Abstract

Aggregation of opinions often results in high decision-making accuracy, owing to the collective intelligence effect. Studies on group decisions have examined the optimum weights for opinion aggregation to maximise accuracy. In addition to the optimum weights of opinions, the impact of the correlation among opinions on collective intelligence is a major issue in collective decision-making. We investigated how individuals should weigh the opinions of others and their own to maximise their accuracy in sequential decision-making. In our sequential decision-making model, each person makes a primary choice, observes his/her predecessors’ opinions, and makes a final choice, which results in the person’s answer correlating with those of others. We developed an algorithm to find casting voters whose primary choices are determinative of their answers and revealed that decision accuracy is maximised by considering only the abilities of the preceding casting voters. We also found that for individuals with heterogeneous abilities, the order of decision-making has a significant impact on the correlation between their answers and their accuracies. This could lead to a counter-intuitive phenomenon whereby, in sequential decision-making, respondents are, on average, more accurate when less reliable individuals answer earlier and more reliable individuals answer later.

## Introduction

Individuals often incorporate others’ opinions into their decision-making with the motivation to improve their decision accuracy [1]. Indeed, extensive studies have revealed the power of opinion aggregation; majority-rule voting or average of opinions by non-experts, for example, can have higher accuracy than an expert’s opinion [2, 3]. The phenomenon wherein the aggregation of non-experts’ opinions can outperform that of an expert is called *collective intelligence* [4, 5].

Various applications of collective intelligence, including future forecasts [6–8], crowdsourcing [9], risk assessments [10], lie-detection [11], medical diagnose [12, 13], and ensemble methods (e.g. random forest) in machine learning [14] have been investigated. Empirical studies have shown that opinion aggregation accuracy can be maximised by assigning equal weights to all opinions offered by individuals with similar problem solving abilities. However, when their abilities vary significantly, the expert rule, wherein only a single or few individuals with high ability contribute to decision-making, performs better [12, 15].

For a binary-choice problem with one correct and one wrong choice, the law of large numbers has mathematically explained the reason behind the higher accuracy of a majority vote compared to an individual’s decision-making [2, 16–20]. Theoretical studies have also revealed that the optimum weight to be given to opinions to maximise the majority vote accuracy is proportional to the log-odds ratio of the individual’s ability [17, 18, 21]. Such optimum weighted majority rule is equivalent to the expert rule when the individuals’ abilities vary significantly and more than half of the weight should be assigned to an individual; this can partially explain the aforementioned empirical findings [12, 15] showing that opinion aggregation with the same weights on individuals is sometimes inferior to the expert rule. In addition to assigning weights to opinions, a group decision accuracy is significantly influenced by the correlation between opinions [2, 22–24]. Although opinion correlation frequently occurs in society, such a correlation between opinions frequently deteriorates collective intelligence by reducing the effective number of independent votes [2, 23, 25].

Our interest lies in assigning weights to opinions that can be correlated. Particularly, we consider the opinion correlation generated by rational individuals attempting to maximise their decision accuracy by incorporating each other’s opinions. Such a situation can naturally occur when individuals decide sequentially rather than simultaneously. In most, if not all, cases, collective decision-making in our society is sequential, where people decide by considering earlier presented opinions and sometimes share their ideas to the public to also affect others who decide later. Previous studies on sequential decision-making have investigated the probability of an individual choosing an option given social information, which is the (relative) number of predecessors supporting the option [26–30]. They also examined the extent to which an individual should consider social information in his/her decision-making to obtain a high decision accuracy, given the estimated abilities of him/herself and others. The strength of such reliance on social information is shown to affect not only an individual’s decision accuracy but also the frequency of information cascade occurring in a group, where most individuals choose the same option under conformity [27, 29, 31]. In these previous studies, however, the heterogeneity in individuals’ abilities and the correlation between individuals’ answers are not sufficiently considered. For example, in social information, while some opinions can be given by experts who decide by themselves, others can be provided by individuals with low abilities who followed others’ opinions to improve their decision accuracy. The optimum weights for accuracy maximisation in sequential decision-making considering such a complicated correlation structure between predecessors’ answers have not been investigated precisely.

In this study, we considered sequential decision-making [29, 31, 32], where individuals’ answers correlated due to the heterogeneity in their abilities and motivation to follow predecessors. The aforementioned weights being proportional to the log-odds ratios of individuals’ abilities are shown to be optimum for independent opinions and are not applicable to correlated opinions. Intuitively, one might think that to properly evaluate and weigh the predecessors’ opinions, one needs to know not only the predecessors’ expressed answers but also the unobservable decision process that led to their answers. Indeed, humans have the ability to infer the inner decision process of others, called the Theory of Mind (ToM) [33–37]. By contrast, this study showed that no such inference is necessary to optimally weigh the predecessors’ opinions by assuming that one’s predecessors also maximise their accuracy.

We proposed a simple algorithm for identifying *casting voters* whose inner primary opinions are directly reflected in their expressed answers. Applying this algorithm to several examples of sequential decision-making, we found that individuals can maximise their performance by optimally weighing casting voters in the predecessors. Such optimum decision-making by individuals can generate opinion correlation between their answers. Accordingly, later responding individuals in sequential decision-making cannot obtain a significant effect of collective intelligence from their predecessors’ answers, where a similar phenomenon has been previously pointed out in the study on the maintenance of opinion diversity [38]. We further showed that the decision order of individuals with heterogeneous credibility significantly affects the correlation strength among their answers, optimal decision, and accuracy. Our results indicated that the expression of opinions by experts at the beginning of sequential decision-making may compromise the accuracy of the later respondents’ answers because such a decision order would motivate respondents to follow the preceding individuals, thus generating correlations between answers used by the later respondents in their decision-making and undermining the collective intelligence effect.

## Methods

### Model

#### Sequential decision-making in a group solving a binary-choice problem

In this study, we considered a binary-choice problem with one correct and one incorrect option; *N* persons answer this problem sequentially. All respondents, except the first, can refer to the answers of earlier respondents, or *antecedents*, in making their decisions. We call this process *sequential decision-making*.

#### Ability and primary choice of a respondent

The ability of the *n*-th respondent, denoted by *p*_*n*_, is defined as the probability of making the correct choice independently. We call such an independent choice the *primary choice*. Let us denote the first respondent’s option in the binary choice as *s* and the other option not selected as *t*. The primary choice of the *n*-th responder, denoted by *X*_*n*_, can be either *s* or *t*, where *X*_*n*_ = *s* or *X*_*n*_ = *t* indicates that the primary choice is identical or opposite to the first respondent’s answer, respectively. We assumed that *p*_*n*_ is between 0.5 and 1; 0.5 is the worst success probability when the decision is made by coin-flipping, and 1 is the best probability when the individual always makes the correct choice. To avoid tie resolving complications, we assumed that the abilities of two different responders differ, even slightly, from each other (i.e. *p*_*n*_ ≠ *p*_*m*_ if *n* ≠ *m*). For the *n*-th individual’s primary choice, the ratio of the probabilities of making a correct choice to an incorrect choice, *p*_*n*_/(1 − *p*_*n*_), is called the odds ratio. We also assumed that for any pair of different subsets *S* and *T*(*S* ≠ *T*) of the set {1, 2, …, *N*} of responders (the numeral represents the order of decision-making), the product of the odds ratios of individuals belonging to each set differs from each other, (∏_*m*∈*S*_ *p*_*m*_/(1 − *p*_*m*_) ≠ ∏_*m*∈*T*_ *p*_*m*_/(1 − *p*_*m*_) to simplify later discussion.

#### Synthesizing primary choice and antecedents’ answers

All respondents, except the first, of sequential decision-making observe the answers given by the antecedents, revise their primary choices by considering the antecedents’ choices, and make their final decisions, which we call *answers*. Therefore, the *n*-th respondent’s answer, denoted by *Y*_*n*_, can be different from his/her primary choice *X*_*n*_, and depends on his/her own primary choice and the antecedents’ answers: *Y*_*n*_ = *Y*_*n*_|*X*_*n*_, *Y*_*n*−1_, *Y*_*n*−2_, ⋯, *Y*_1_. We assumed that each primary choice is not shared with other individuals, while each answer can be observed by them. Similar to primary choice, the value of the answer is either *s* or *t*(*Y*_*n*_ = *s* or *Y*_*n*_ = *t*) depending on whether the answer is identical to that of the first respondent.

#### Optimal answers maximizing conditional performances

Using these definitions, we describe the detailed process of sequential decision-making. The first respondent decides and answers independently, relying only on his/her primary choice (*Y*_1_ = *X*_1_ = *s*). Therefore, the probability of his/her answer being correct equals his/her ability *p*_1_. For *n* ≥ 2, the *n*-th respondent observes the answer(s) of the antecedent(s). We assumed that the *n*-th respondent knows his/her individual and antecedents’ abilities (*p*_1_, *p*_2_, ⋯, *p*_*n*_), antecedents’ answers (*Y*_1_, *Y*_2_, ⋯, *Y*_*n*−1_), and his/her own primary choice *X*_*n*_. The respondent then attempts to maximise the correct option selection probability under these conditions. We call this maximised probability of a respondent to answer correctly *conditional performance* and this decision-making method the *optimal behaviour*. Although optimum decision-making for group performance maximisation is interesting, we limit our discussion to the maximisation of individual performance conditional to antecedents’ answers in this study. We assumed that each individual knows that his/her antecedents behave optimally. We clarify our assumption on the information available for the *n*-th respondent to make the hierarchy-based optimal decision similar to that of ToM [33–35]. The first respondent makes an independent choice. We call such an optimum choice the level-0 optimum. The second respondent relies on both his/her own primary choice and the level-0 optimum answer to make his/her optimum choice, which we call a level-1 optimum. This procedure continues recursively. The *n*-th respondent relies on his/her own primary choice and *n* − 1 antecedent optimum answers, i.e., from a level-(*n* − 2) optimum answer of the (*n* − 1)-th respondent to a level-0 optimum answer of the first respondent, to make his/her optimum decision called a level-(*n* − 1) optimum. Individual respondents do not need to know such a hierarchical information structure when making their decisions (they only need to know the sequence of answers taken by the antecedents and their presumed or known abilities); however, this helps to clarify the knowledge structure assumed in this study.

Optimal behaviour can be regarded as a weighted majority vote in the following sense: answering an option that leads to a greater conditional performance can be realised when sufficiently high weights are assigned to individuals (antecedents and respondent) who choose the option based on the antecedents’ given answers and abilities, as shown in the Results section.

#### Mean performance

As individuals’ primary choices are made randomly according to their abilities, the answers of antecedents who behave optimally also form a random sequence in each sequential decision-making process. With a fixed set of abilities and the answering orders of antecedents, we defined the *mean performance* of a respondent as the mean of the conditional performance over possible antecedents’ answers and his/her primary choice. The nature of sequential decision-making can be characterised by the mean performance of each respondent, i.e., the expectation of the correct answer from the respondent. We illustrated this through the sequential decision-making of three individuals and individuals with the same abilities (the section ‘Specific cases’).

### Optimum weights in simultaneous decision-making

#### Weighted majority vote

To discuss the implications of optimal behaviour in sequential decision-making, we need to briefly summarise the previous results of optimally weighted majority vote for maximising *simultaneous decision-making* accuracy [16–18]. In the simultaneous decision-making of a binary choice, individuals vote their primary choices independently, which are then aggregated with their weights. More precisely, the outcome of the simultaneous decision-making of *N* individuals is to adopt the option that receives the highest weighted vote. We denote by *S* and *T* the set of indices of individuals who choose the alternatives *s* and *t*, respectively, where *S* ∪ *T* = {1, 2, ⋯, *N*}.

#### Likelihood of opinion distribution

We denoted the weight assigned to an individual *n* by *w*_*n*_ and normalised it as $\sum_{n=1}^{N}w_{n}=1$. The aggregated votes to *s* and *t* are *W*(*S*) = ∑_*n*∈*S*_ *w*_*n*_ and *W*(*T*) = ∑_*n*∈*T*_ *w*_*n*_, respectively. If *s* is correct, the probability that this opinion distribution is obtained is *L*(*S*) = *P*(*S*)*Q*(*T*), where *P*(*S*) = ∏_*n*∈*S*_ *p*_*n*_ and *Q*(*T*) = ∏_*n*∈*T*_ (1 − *p*_*n*_) are the probabilities that all answers of individuals in group *S* are correct and in group *T* are incorrect, respectively. *p*_*n*_ denotes the ability of the *n*-th individual (*n* = 1, 2, ⋯, *N*). *L*(*S*) is also regarded as the likelihood that *s* is correct, given the opinion distribution with *S* and *T*. Similarly, the probability that this opinion distribution is obtained if *t* is correct, or the likelihood that *t* is correct given the opinion distribution, is *L*(*T*) = *P*(*T*)*Q*(*S*).

#### Optimum weights

Here, we discuss the weight assignment to responders such that the option that is selected by the weighted majority vote should always have a higher probability of being correct than the other, under the given vote distribution (sets *S* and *T* for options *s* and *t*, respectively) among responders. This is possible as follows [16, 17] (S.1.1 in S1 Text). Suppose that option *s* is selected by the weighted majority vote, i.e., *W*(*S*) = ∑_*n*∈*S*_ *w*_*n*_ > *W*(*T*) = ∑_*n*∈*T*_ *w*_*n*_. The probability that the observed distribution of opinions occurs under the abilities *p*_1_, ⋯, *p*_*n*_ of respondents is *L*_*s*_ = ∏_*n*∈*S*_ *p*_*n*_ ∏_*n*∈*T*_ (1 − *p*_*n*_) if *s* is correct and *L*_*t*_ = ∏_*n*∈*S*_(1 − *p*_*n*_) ∏_*n*∈*T*_ *p*_*n*_ if *t* is correct. The option selected by the weighted majority vote has a higher probability of being correct if *L*_*s*_ > *L*_*t*_ or ∏_*n*∈*S*_ [*p*_*n*_/(1 − *p*_*n*_)] > ∏_*n*∈*T*_ [*p*_*n*_/(1 − *p*_*n*_)]. Therefore, the optimum weights *w*_1_, ⋯, *w*_*n*_ must have following the property:

$$\underset{n\in S}{\sum }\;w_{n}>\underset{n\in T}{\sum }\;w_{n}\;\text{implies}\;\text{that}\;\underset{n\in S}{\prod }\frac{p_{n}}{1-p_{n}}>\underset{n\in T}{\prod }\frac{p_{n}}{1-p_{n}}.$$ (1)

A property identical to (1) should be satisfied with the roles reversed between *S* and *T*. One way to realise this property is to assign the weight of each respondent to the logarithmic odds ratio of his/her credibility, as shown below.

#### Log-odds ratio as an optimum weight

It is well known that if the abilities vary between individuals, the log-odds ratios of individual abilities give a set of optimum weights that maximise the weighted majority vote accuracy [17, 18, 21]. Indeed, we can show that by assigning a weight to an individual as

$$w_{n}\propto r_{n}^{*}=\text{log}\;\frac{p_{n}}{1-p_{n}},$$

the above relationship (1) for optimality always holds because the inequality on the right-hand side of (1) is written as the summation of $r_{n}^{*}$ as

$$\underset{n\in S}{\prod }\frac{p_{n}}{1-p_{n}}>\underset{n\in T}{\prod }\frac{p_{n}}{1-p_{n}}\Leftrightarrow \underset{n\in S}{\sum }\;r_{n}^{*}>\underset{n\in T}{\sum }\;r_{n}^{*}.$$

**Fig 1:**
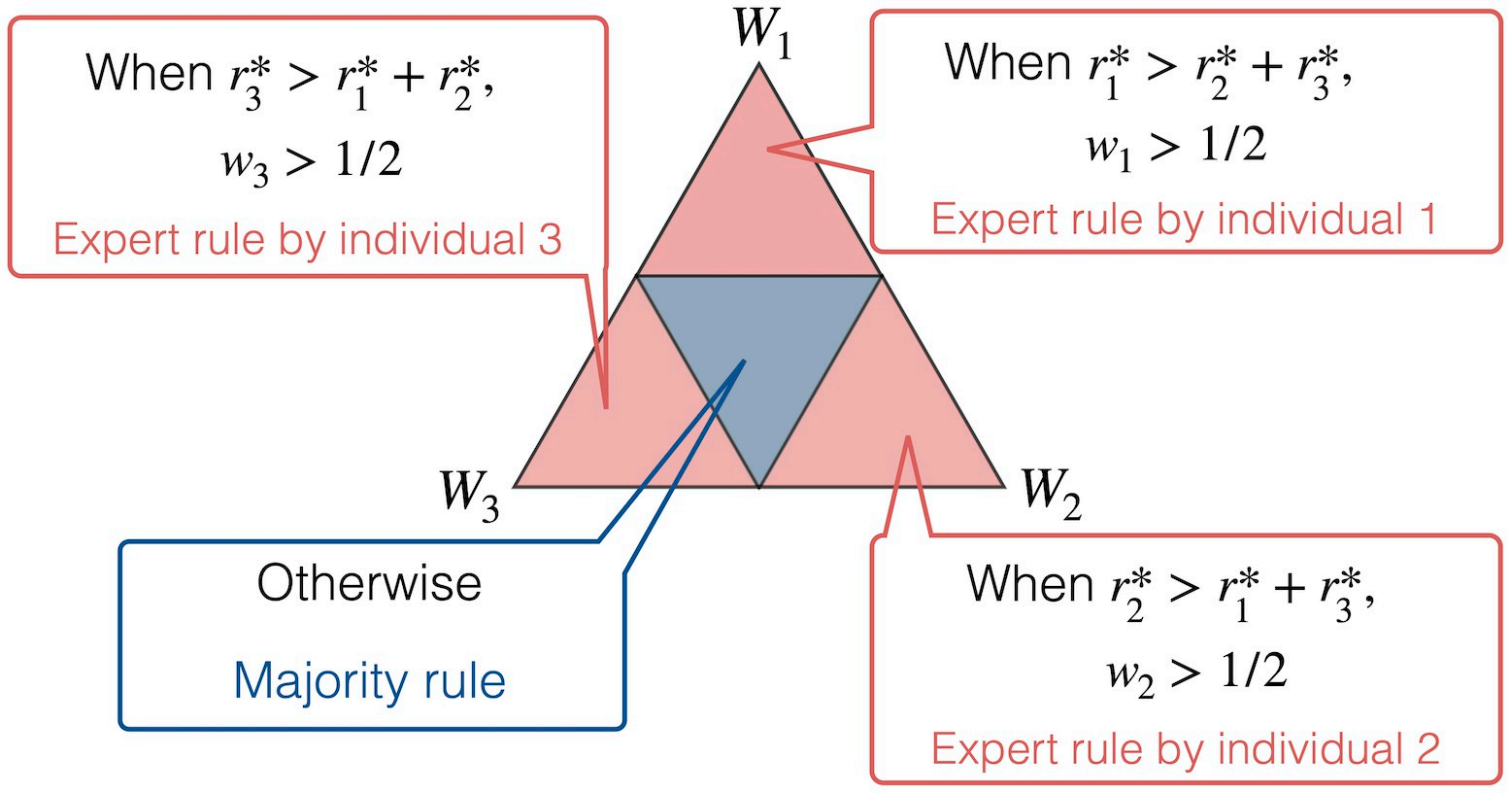
Summary of the region of optimum weights *w*_*n*_ for individual *n* (*n* = 1, 2, 3) in the simultaneous decision-making involving three individuals. The largest triangle exhibits the 2-simplex {*w* = (*w*_1_, *w*_2_, *w*_3_); *w*_1_ + *w*_2_ + *w*_3_ = 1}, where *W*_1_, *W*_2_, and *W*_3_ denote (1,0,0), (0,1,0), and (0,0,1), respectively. Each smaller triangle exhibits the region of optimum weights when each inequality for $r_{1}^{*}$, $r_{2}^{*}$, and $r_{3}^{*}$ holds. For example, when the ability of individual 1 is so high that $r_{1}^{*}>r_{2}^{*}+r_{3}^{*}$ is satisfied, the optimum weights should satisfy *w*_1_ > 0.5, i.e., *w*_2_ + *w*_3_ < 0.5, which corresponds to the upper red triangle, and these weights correspond to the expert rule governed by individual 1.

While $r_{n}^{*}$ provides a simple solution for the efficient majority vote of agents with different abilities, it is not a unique way to weigh agents optimally, as Shapley and Grofman [18] noted, and is shown in Fig 1.

Although quite useful, these results are applicable only when the respondents make their decisions *simultaneously*, where there is no room for the opinions to correlate with each other. In *sequential* decision-making, the opinions of respondents are no longer independent, as each respondent refers to the antecedents’ answers when he/she makes a decision. Therefore, we need to develop a theory for the optimum behaviour of each respondent in sequential decision-making that maximises conditional performance, as discussed in the following sections.

## Results

### Casting vote

First, let us consider the second respondent’s optimal decision. By definition, the first respondent decided based only on his/her primary choice. The second individual decides using the first individual’s answer *Y*_1_ and his/her primary choice *X*_2_, knowing that his/her and the first individual’s abilities are *p*_2_ and *p*_1_, respectively. Since the primary choice *X*_2_ is independent of answer *Y*_1_, we can apply the optimum weight rule of simultaneous decision-making discussed in the section ‘Optimum weights in simultaneous decision-making’, to find the optimal behaviour of the second individual. If *p*_1_ > *p*_2_, the second individual should always choose the same answer as the first individual (*s*) irrespective of his/her primary choice. By contrast, if *p*_1_ < *p*_2_, the second individual should always adopt his/her primary choice, irrespective of the answer of the first individual (S.1.2 in S1 Text). Particularly, his/her primary choice determines the majority vote in the binary-choice problem in her inner process of finding an optimum answer. We call this primary choice *casting vote*. An important point is that an individual’s answer is independent of the antecedents’ answers if the individual’s primary choice is the casting vote.

The third respondent can evaluate the likelihood of each choice being correct from his/her abilities and the observed answers *Y*_1_ and *Y*_2_ of the two antecedents. As he/she knows the abilities *p*_1_ and *p*_2_ of the antecedents and that the second individual behaved optimally, she can evaluate the probability of observing these answers when *Y*_1_ = *s* is correct, as well as the corresponding probability when alternative *t*, not taken by the first, is correct, as follows. If *p*_1_ < *p*_2_, *Y*_1_ and *Y*_2_ must be independent of each other, because the primary choice of the second individual is the casting vote. Therefore, if the answers of the first and second respondents are the same (*Y*_1_ = *Y*_2_ = *s*), then the likelihood that the answer of the first, *Y*_1_ = *s*, is correct is *p*_1_*p*_2_, whereas the likelihood that the answer *t* is correct is (1 − *p*_1_)(1 − *p*_2_). However, if *Y*_1_ = *s* and *Y*_2_ = *t*, the likelihoods of *s* and *t* being correct are *p*_1_(1 − *p*_2_) and (1 − *p*_1_)*p*_2_, respectively. By contrast, if *p*_1_ > *p*_2_, their answers must always be the same (*Y*_1_ = *Y*_2_ = *s*) because the second respondent must follow the first answer; thus, the likelihoods of *s* and *t* being correct are *p*_1_ and 1 − *p*_1_, respectively. *p*_2_ does not affect these likelihoods when *p*_1_ > *p*_2_ because the second individual adopts the same answer as the first, regardless of his/her primary choice. We can then inductively show the following theorem, the *casting vote theorem*, for the probabilities of observing the antecedents’ answers (the formal proof of which is shown in Section S.2.1 of S1 Text).

Let *Y*_1_, ⋯, *Y*_*n*−1_ be the answers and *p*_1_, …, *p*_*n*−1_ be the abilities of *n* − 1 antecedents of the *n*-th respondent in sequential decision-making (2 ≤ *n* ≤ *N*) as described in Model section. Let *S*_*n*−1_, *T*_*n*−1_, and *R*_*n*−1_ be subsets of antecedents who were casting voters and answered *s*, casting voters and answered *t*, and non-casting voters whose primary choices were not casting votes. By definition, the first respondent, 1, is included in *S*_*n*−1_. Sets *S*_*n*−1_, *T*_*n*−1_, and *R*_*n*−1_ are mutually exclusive and jointly cover all respondents from the first to (*n* − 1)-th respondents: *S*_*n*−1_ ∪ *T*_*n*−1_ ∪ *R*_*n*−1_ = {1, 2, ⋯, *n* − 1}. Each respondent is assumed to be rational in the sense that he/she chooses her answer to maximise the probability of answering correctly under the condition shown in Model section.

#### Theorem

(Casting vote theorem)

1. (Alternative) The optimal behaviour of the *n*-th individual is either i) to provide his/her primary choice as the answer regardless of his/her antecedents’ answers or ii) to disregard his/her own primary choice and answer based only on his/her antecedents’ answers. Particularly, his/her own primary choice is either perfectly adopted or ignored in the optimal decision, with no partial incorporation of his/her primary choice.
2. (Likelihoods) The probability of observing the given subsets *S*_*n*−1_ of casting voters who chose *s*, *T*_*n*−1_ of casting voters who chose *t*, and *R*_*n*−1_ of non-casting voters is given by $P\left(S_{n-1}\right)Q\left(T_{n-1}\right)=\prod_{m\in S_{n-1}}p_{m}\prod_{m\in T_{n-1}}\left(1-p_{m}\right)$ when the first respondent answered correctly (i.e. if *s* was correct), and $Q\left(S_{n-1}\right)P\left(T_{n-1}\right)=\prod_{m\in S_{n-1}}\left(1-p_{m}\right)\prod_{m\in T_{n-1}}p_{m}$ when *t* was correct. Particularly, these probabilities (likelihoods) are solely determined by the abilities of the casting voters.

An interpretation of the casting vote theorem is that the *n*-th individual can know and use the information on the primary choice of the *m*-th individual (*m* < *n*) only when the *m*-th individual is a casting voter, i.e., *m* ∈ *S* ∪ *T*; in this case, *Y*_*m*_ = *X*_*m*_ always holds.

### Optimal behaviour

Now, we describe how the optimum choice of the *n*-th respondent is made using the casting vote theorem (see S.1.1 and S.2.1 in S1 Text for detail). The criterion for the optimum choice differs slightly depending on whether the primary choice is identical to that of the first respondent. Hence, we first consider the case where the primary choice of the *n*-th respondent is identical to that of the first respondent *s* (*X*_*n*_ = *s*). The likelihood of *s* and *t* being correct are *P*(*S*_*n*−1_)*Q*(*T*_*n*−1_)*p*_*n*_ and *Q*(*S*_*n*−1_)*P*(*T*_*n*−1_)(1 − *p*_*n*_), respectively, according to the casting vote theorem, given the antecedents’ answers included in either *S*_*n*−1_, *T*_*n*−1_ or *R*_*n*−1_. Therefore, the optimal answer of the *n*-th respondent is *s* if the likelihood ratio *P*(*S*_*n*−1_)*Q*(*T*_*n*−1_)*p*_*n*_/(*Q*(*S*_*n*−1_)*P*(*T*_*n*−1_)(1 − *p*_*n*_)) > 1, and *t* otherwise. These conditions can be written using the sum of the log-odds ratio, $r_{n}^{*}=\text{log}\left[p_{n}/\left(1-p_{n}\right)\right]$, of individual credibility, which plays a key role in simultaneous decision-making, as discussed in the section ‘Optimum weights in simultaneous decision-making’. Since the logarithm of the above likelihood ratio can be rewritten as $\text{log}\left[P\left(S_{n-1}\right)Q\left(T_{n-1}\right)p_{n}/\left(Q\left(S_{n-1}\right)P\left(T_{n-1}\right)\left(1-p_{n}\right)\right)\right]=r_{n}^{*}+\sum_{m\in S_{n-1}}r_{m}^{*}-\sum_{m\in T_{n-1}}r_{m}^{*}$, where log[*P*(*X*)/*Q*(*X*)] = 0 if set *X* is empty, the optimal answer of the *n*-th respondent when *X*_*n*_ = *s* is

$${\left.Y_{n}\right|}_{X_{n}=s}=\left\{\begin{array}{ll} s, & \text{if}\;\;\;r_{n}^{*}>\sum_{m\in T_{n-1}}\;r_{m}^{*}-\sum_{m\in S_{n-1}}\;r_{m}^{*},\\ t, & \text{if}\;\;\;r_{n}^{*}<\sum_{m\in T_{n-1}}\;r_{m}^{*}-\sum_{m\in S_{n-1}}\;r_{m}^{*}. \end{array}\right.$$ (2)

Similarly, if the primary choice of the *n*-th respondent is *t*(*X*_*n*_ = *t*), the optimal answer of the *n*-th respondent when *X*_*n*_ = *t* is

$${\left.Y_{n}\right|}_{X_{n}=t}=\left\{\begin{array}{ll} s, & \text{if}\;\sum_{m\in S_{n-1}}\;r_{m}^{*}-\sum_{m\in T_{n-1}}\;r_{m}^{*}>r_{n}^{*},\\ t, & \text{if}\;\sum_{m\in S_{n-1}}\;r_{m}^{*}-\sum_{m\in T_{n-1}}\;r_{m}^{*}<r_{n}^{*}. \end{array}\right.$$ (3)

Note that, as expected from the casting vote theorem, the optimal decisions are independent of the decisions made by non-casting voters (*R*_*n*−1_); thus, non-casting voters have no contribution to the optimum decision criteria in (2) and (3).

The next task is to determine to which subset (*S*_*n*_, *T*_*n*_, or *R*_*n*_) the focal *n*-th respondent will belong. The primary choice of the *n*-th respondent is a casting vote if $|\sum_{m\in S_{n-1}}\;r_{m}^{*}-\sum_{m\in T_{n-1}}\;r_{m}^{*}|<r_{n}^{*}$. This corresponds to the case where the liabilities for the decisions made by antecedent casting voters who chose *s* and *t* are comparable, i.e. the difference between the summations of log-odds ratios is less than $r_{n}^{*}$, the log-odds ratio of the primary choice of focal respondent *n*. In this case, the *n*-th respondent is a casting voter who answered *s* (*n* ∈ *S*_*n*_) and *t* (*n* ∈ *T*_*n*_) if his/her primary choices were *s* (*X*_*n*_ = *s*) and *t* (*X*_*n*_ = *t*), respectively. By contrast, $\text{if}|\sum_{m\in S_{n-1}}\;r_{m}^{*}-\sum_{m\in T_{n-1}}\;r_{m}^{*}|>r_{n}^{*}$, the *n*-th respondent is a non-casting voter.

The two procedures described in the preceding two paragraphs provide a recursive algorithm to determine the optimum decisions in sequential decision-making, which are summarised in Table 1. We can recursively determine, without any uncertainty, whether each, say the *n*-th respondent, is a casting voter by using the abilities (*p*_1_, …, *p*_*n*_) and the sets *S*_*n*−1_ and *T*_*n*−1_. If the *n*-th individual is a casting voter (*n* ∈ *S*_*n*−1_ ∪ *T*_*n*−1_), his/her primary choice is always observed by the others through his/her answer. When the *n*-th individual is a non-casting voter (*n* ∈ *R*_*n*−1_), while his/her primary choice cannot be known by the others, the latter can behave optimally without using his/her primary choice.

**Table 1:** Algorithm for optimal decision determination in sequential decision-making. See text for the definitions of subsets *S*, *T*, and *R*, the log-odds ratio of the *n*-th respondent’s ability, $r_{n}^{*}$, and binary-choice alternatives, *s* and *t*. *ϕ* is an empty set.

1: *S* = {1}, *T* = ∅, *R* = ∅ 2: **for** 2 ≤ *n* ≤ *N* **do** 3: **if $\sum_{m\in S}\;r_{m}^{*}-\sum_{m\in T}\;r_{m}^{*}>r_{n}^{*}$ then** 4: *R* ← *R* ∪ {*n*}, *Y*_*n*_ = *s* 5: **else if $\sum_{m\in T}\;r_{m}^{*}-\sum_{m\in S}\;r_{m}^{*}>r_{n}^{*}$ then** 6: *R* ← *R* ∪ {*n*}, *Y*_*n*_ = *t* 7: **else if $|\sum_{m\in S_{n-1}}\;r_{m}^{*}-\sum_{m\in T_{n-1}}\;r_{m}^{*}|<r_{n}^{*}$ then** 8: **if** *X*_*n*_ = *s* **then** 9: *S* ← *S* ∪ {*n*}, *Y*_*n*_ = *s* 10: **else** 11: *T* ← *T* ∪ {*n*}, *Y*_*n*_ = *t* 12: **end if** 13: **end if** 14: *S*_*n*_ = *S*, *T*_*n*_ = *T*, *R*_*n*_ = *R* 15: **end for**

By optimal behaviour, each respondent, say the *n*-th, can always select the choice with a higher likelihood of being correct. In this way, he/she maximises his/her conditional performance *π*_*n*_(*S*_*n*−1_, *T*_*n*−1_, *R*_*n*−1_, *X*_*n*_) based on his/her primary choice *X*_*n*_ and the distribution (*S*_*n*−1_, *T*_*n*−1_, *R*_*n*−1_) of the antecedents’ answer. Conditional performance *π*_*n*_(*S*_*n*−1_, *T*_*n*−1_, *R*_*n*−1_, *X*_*n*_) is then obtained by dividing the joint probability of the optimal choice being correct and the opinion distribution of the antecedents’ answers and primary choice being (*S*_*n*−1_, *T*_*n*−1_, *R*_*n*−1_, *X*_*n*_) by the probability of the opinion distribution being (*S*_*n*−1_, *T*_*n*−1_, *R*_*n*−1_, *X*_*n*_):

$$\pi_{n}\left(S_{n-1},T_{n-1},R_{n-1},X_{n}\right)=\left\{\begin{array}{ll} \frac{\text{max}\left\{P\left(S_{n-1}\right)Q\left(T_{n-1}\right)p_{n},\;Q\left(S_{n-1}\right)P\left(T_{n-1}\right)\left(1-p_{n}\right)\right\}}{P\left(S_{n-1}\right)Q\left(T_{n-1}\right)p_{n}+Q\left(S_{n-1}\right)P\left(T_{n-1}\right)\left(1-p_{n}\right)}, & \text{if}\;X_{n}=s,\\ \frac{\text{max}\left\{P\left(S_{n-1}\right)Q\left(T_{n-1}\right)\left(1-p_{n}\right),\;Q\left(S_{n-1}\right)P\left(T_{n-1}\right)p_{n}\right\}}{P\left(S_{n-1}\right)Q\left(T_{n-1}\right)\left(1-p_{n}\right)+Q\left(S_{n-1}\right)P\left(T_{n-1}\right)p_{n}}, & \text{if}\;X_{n}=t. \end{array}\right.$$ (4)

### Specific cases

#### Three persons with arbitrary abilities

Here, we consider a specific case of sequential decision-making by only three individuals and analyse their optimal behaviours in greater detail (the complete calculation is in S.2.2 in S1 Text).

If an individual is the first to answer, he/she bases her response only on his/her primary choice. Therefore, his/her performance always equals ability *p*_1_. By the definition of *s* and *S*_*n*_, 1 is included in set *S*_1_. For the second respondent, the preceding vote distribution is always the same: *S*_*n*_ = {1}, *T*_1_ = ∅, and *R*_1_ = ∅, where ∅ denotes the empty set. Therefore, his/her conditional performance, *π*_2_(*S*_1_, *T*_1_, *R*_1_, *X*_2_), is equivalent to his/her mean performance E[*π*_2_(*S*_1_, *T*_1_, *R*_1_, *X*_2_)]. The optimal behaviours of the second and third respondents markedly differ depending on which of the first and second’s abilities is larger, as shown in the following.

First, if *p*_2_ > *p*_1_, that is, $r_{2}^{*}>r_{1}^{*}$, the second respondent should answer his/her primary choice regardless of the first respondent’s answer, as discussed in the section ‘Casting vote’. Therefore, 2 ∈ *S*_2_ if *X*_2_ = *s* and 2 ∈ *T*_2_ if *X*_2_ = *t*. The conditional and mean performances of the second respondent are then equal to his/her ability: *π*_2_(*S*_1_, *T*_1_, *R*_1_, *X*_2_) E[*π*_2_(*S*_1_, *T*_1_, *R*_1_, *X*_2_)] = *p*_2_. The preceding vote distribution for the third respondent is either (*S*_2_, *T*_2_, *R*_2_) = ({1,2}, ∅, ∅) or (*S*_2_, *T*_2_, *R*_2_) = ({1}, {2}, ∅). Note that, in either case, both the first and second respondents are casting voters. Taking them together with the third respondent’s primary choice, he/she has three independent votes before deciding on his/her answer. Therefore, the optimal answer of the third respondent is the same as the optimal decision in the simultaneous decision-making involving three individuals discussed in the section ‘Optimum weights in simultaneous decision-making’. Particularly, i) if $r_{3}^{*}>r_{1}^{*}+r_{2}^{*}$, the third respondent takes his/her primary choice as his/her answer, ii) if $r_{2}^{*}>r_{1}^{*}+r_{3}^{*}$, the third respondent follows the answer from the second respondent, and iii) if neither is the case, the third respondent follows the unweighted majority of the three votes, two answers by the first and second respondents and his/her primary choice. Note that no such case exists with $r_{1}^{*}>r_{2}^{*}+r_{3}^{*}$ under our assumption of *p*_2_ > *p*_1_. The mean performances of the third respondent are *p*_3_, *p*_2_, and *p*_1_*p*_2_*p*_3_ + (1 − *p*_1_)*p*_2_*p*_3_ + *p*_1_(1 − *p*_2_)*p*_3_ + *p*_1_*p*_2_(1 − *p*_3_)(≔ *M*) for cases i), ii), and iii), respectively.

**Fig 2:**
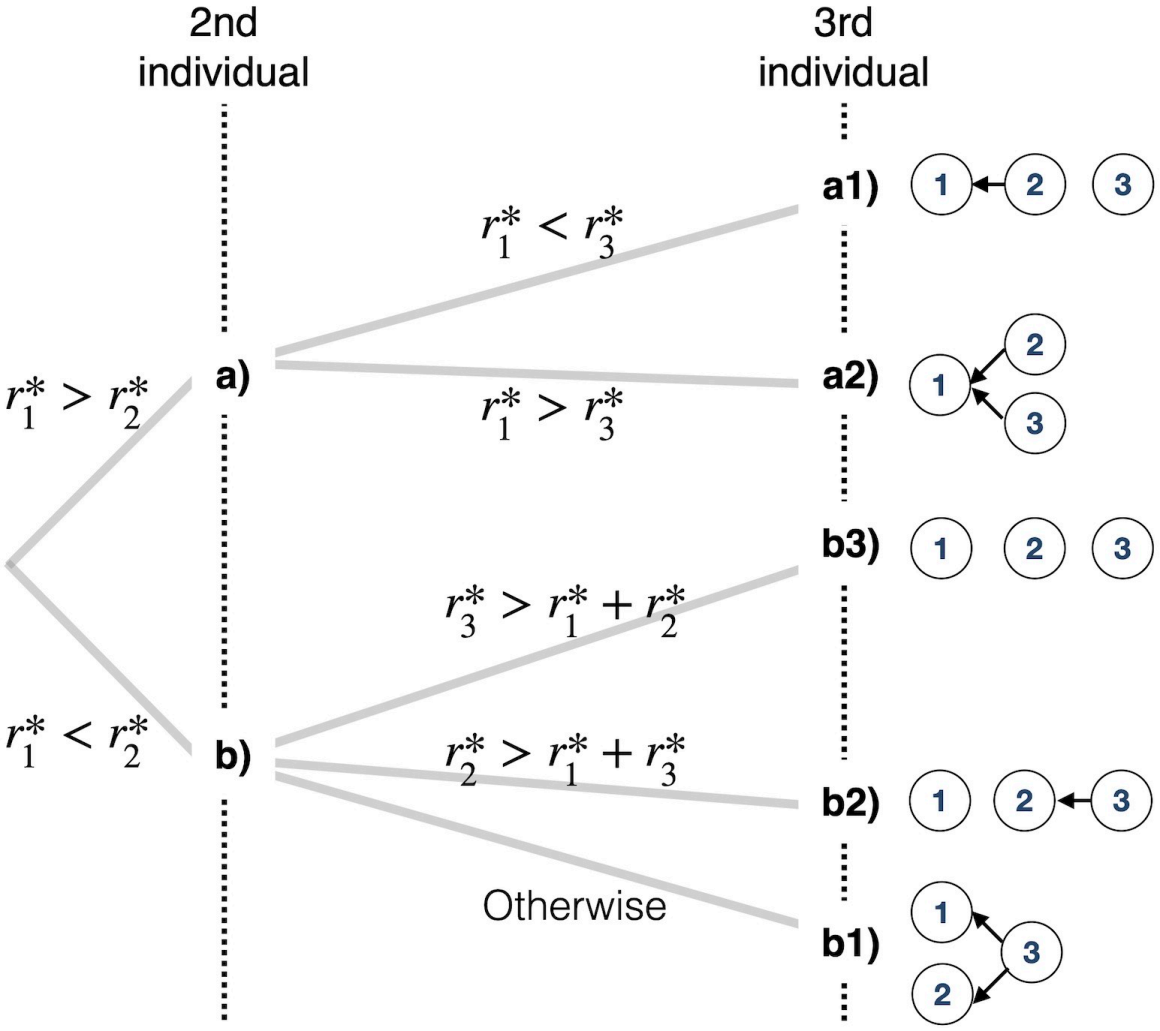
Optimal behaviours of the second and third individuals. The diagram on the right-hand side shows the resulting influential relationship among three individuals by the arrows, each pointing to the individual that one follows. For example, the top diagram (a1) reveals that the second individual incorporates the answer by the first individual, and the third individual decides without referring to others.

Second, if the first respondent has a higher ability than the second (*p*_1_ > *p*_2_, i.e. $r_{1}^{*}>r_{2}^{*}$), the second respondent should always follow answer *s* by the first irrespective of his/her primary choice (see the section ‘Casting vote’), i.e., his/her primary choice is not a casting vote (2 ∈ *R*_2_). The second respondent’s conditional and mean performances are *p*_1_, which is greater than accuracy *p*_2_ of an independent decision. Unlike in the previous case, the answers of the first and second respondents are no longer independent of each other. The third respondent then always sees the same preceding vote distribution: (*S*_2_, *T*_2_, *R*_2_) = ({1},∅,{2}). If the primary choice *X*_3_ is *s*, then by applying the rule in Eq (2), the third respondent should answer *s*. If *X*_3_ = *t*, we apply the rule in Eq (3) to see that he/she should answer *s* when $r_{1}^{*}>r_{3}^{*}$, i.e., *p*_1_ > *p*_3_ and answer *t* when $r_{1}^{*}<r_{3}^{*}$, i.e., *p*_1_ < *p*_3_. The third individual’s mean performance is *p*_1_ and *p*_3_ when *p*_1_ > *p*_3_ and *p*_1_ < *p*_3_, respectively. The optimal behaviour of the three-individual sequential decision-making is summarised in Fig 2.

**Fig 3:**
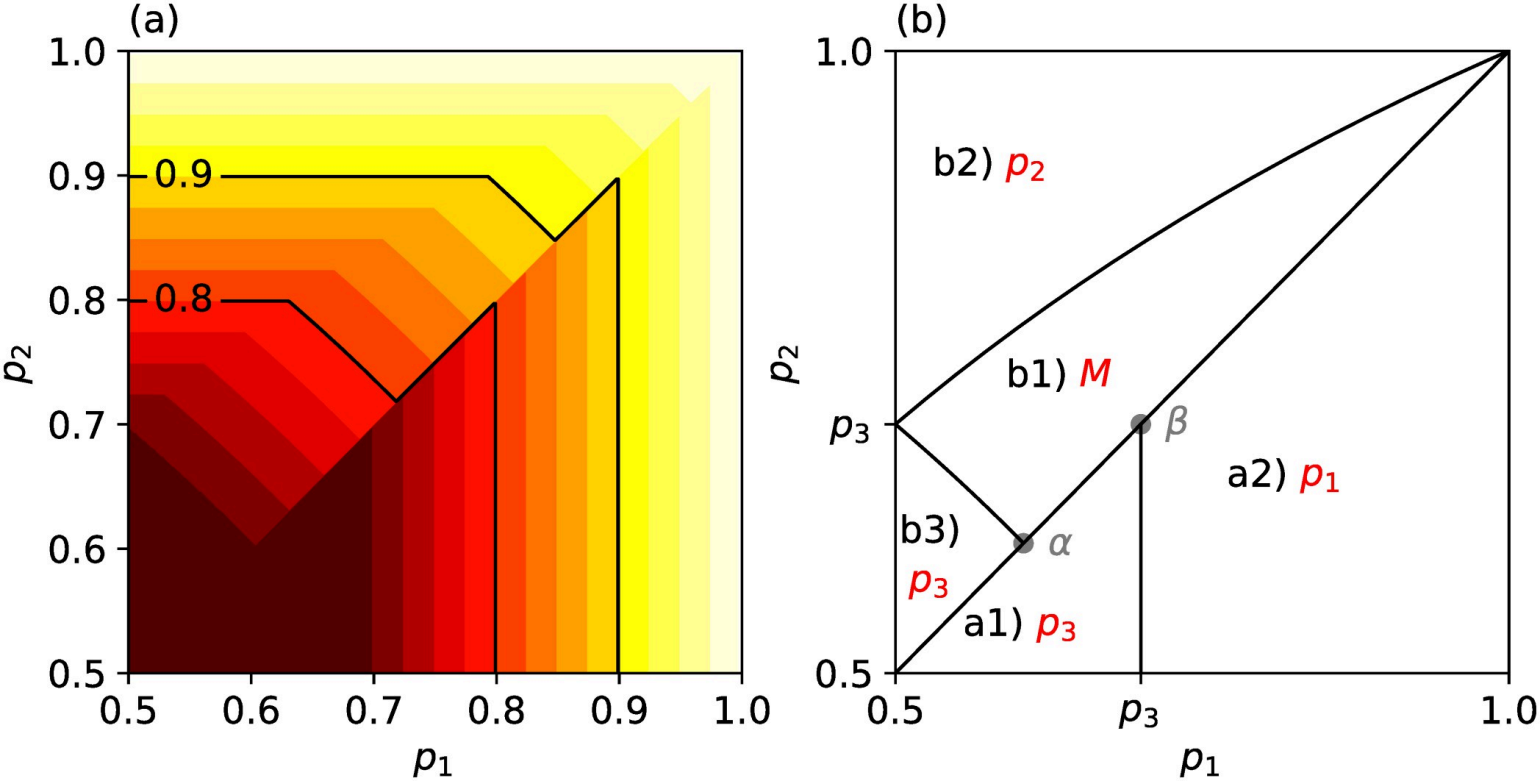
Mean performance of the third individual E[π_3_(*S*_2_, *T*_2_, *R*_2_, *X*_3_)] in sequential decision-making. (a) E[π_3_(*S*_2_, *T*_2_, *R*_2_, *X*_3_)] in sequential decision-making when *p*_3_ = 0.7 is exhibited by contours. The horizontal and the vertical axes show the abilities *p*_1_ and *p*_2_ of the first and second individuals, respectively. The brighter colour stands for the higher mean performance. (b) E[π_3_(*S*_2_, *T*_2_, *R*_2_, *X*_3_)] is shown by red text. The horizontal and vertical axes are the same as that in panel (a). Each region corresponds to each case of a1) to b3) in Fig 2. The coordinates of *α* and *β* are $\sqrt{p_{3}}/\left(\sqrt{p_{3}}+\sqrt{1-p_{3}}\right),\sqrt{p_{3}}/\left(\sqrt{p_{3}}+\sqrt{1-p_{3}}\right)$ and (*p*_3_, *p*_3_), respectively.

Fig 3 shows the mean performance E[*π*_3_] = E[*π*_3_(*S*_2_, *T*_2_, *R*_2_, *X*_3_)] of the third individual when *p*_3_ = 0.7 by its contours and phase diagram. In the contours of the mean performance (Fig 3(a)), we observe that E[*π*_3_] drops significantly at the diagonal line showing *p*_1_ = *p*_2_ from the region of *p*_1_ < *p*_2_ to that of *p*_1_ > *p*_2_. Under the diagonal line (*p*_1_ > *p*_2_), there is no region in which E[*π*_3_] takes the value of *M*, which is the accuracy of the simultaneous unweighted majority vote of three individuals. The value of *M* is greater than *p*_1_ when *p*_1_(1 − *p*_2_)(1 − *p*_3_) < (1 − *p*_1_)*p*_2_*p*_3_ is satisfied or when the first individual is not expert enough for his/her log-odds to outperform the sum of the other two ($r_{1}^{*}<r_{2}^{*}+r_{3}^{*}$). This is because

$$\begin{array}{ll} p_{1} & =p_{1}p_{2}p_{3}+p_{1}p_{2}\left(1-p_{3}\right)+p_{1}\left(1-p_{2}\right)p_{3}+p_{1}\left(1-p_{2}\right)\left(1-p_{3}\right)\\ & <p_{1}p_{2}p_{3}+p_{1}p_{2}\left(1-p_{3}\right)+p_{1}\left(1-p_{2}\right)p_{3}+\left(1-p_{1}\right)p_{2}p_{3}=M. \end{array}$$ (5)

Similarly, *M* is greater than *p*_3_ when the third individual is not expert. Therefore, in the inner region above the diagonal line, where the first individual is not expert and the first and second are not that much inferior to the third, the mean performance of the third respondent dramatically changes according to whether or not *p*_1_ > *p*_2_ because the mean performance of the third cannot be *M* when *p*_1_ > *p*_2_.

#### Arbitrary number of persons with the same ability

In this section, we consider the optimal behaviour in the sequential decision-making involving an arbitrary number of individuals having the same ability *p* (see S.2.3 in S1 Text for detailed derivations of the results shown below). The criteria of Eqs (2) and (3) for the optimal answer *Y*_*n*_ of the *n*-th respondent is simplified for *p*_1_ = *p*_2_ = ⋯ = *p*_*N*_ = *p* as follows. If his/her primary choice is the same as that of the first respondent (*X*_*n*_ = *s*),

$${\left.Y_{n}\right|}_{X_{n}=s}=\left\{\begin{array}{ll} s, & \text{if}\;\left(\left|S_{n-1}\right|-\left|T_{n-1}\right|+1\right)r^{*}\geq 0,\\ t, & \text{if}\;\left(\left|S_{n-1}\right|-\left|T_{n-1}\right|+1\right)r^{*}<0, \end{array}\right.$$ (6)

where |*S*_*n*−1_| and |*T*_*n*−1_| denote the number of casting voters who answered *s* and *t*, respectively, out of *n* − 1 antecedents (|*S*_*n*−1_| + |*T*_*n*−1_| ≤ *n* − 1) and r* = log[*p*/(1 − *p*)], where |*X*| denotes the number of elements in set *X*. In addition, we assumed that if the equalities in Eq (6) hold under the conditions of their odds ratios so that either choice is equally likely to be true, the *n*-th respondent selects his/her primary choice. As r* is positive by assumption (*p* > 0.5), Eq (6) is equivalent to

$${\left.Y_{n}\right|}_{X_{n}=s}=\left\{\begin{array}{ll} s, & \text{if}\;\left|S_{n-1}\right|+1\geq \left|T_{n-1}\right|,\\ t, & \text{if}\;\left|S_{n-1}\right|+1<\left|T_{n-1}\right|. \end{array}\right.$$ (7)

In other words, he/she should answer *s* if the sum of his/her primary vote (*X*_*n*_ = *s*) and the votes to *s* by preceding casting voters is equal to or greater than the votes to *t* by preceding casting voters. By contrast, he/she should answer *t* if, even with his/her primary vote to *s*, the sum of the votes to *s* by him/her and preceding casting voters is less than the votes to *t* by preceding casting voters. Similarly, if the *n*-th respondent’s primary choice is *t*(*X*_*n*_ = *t*), the optimal answer should be

$${\left.Y_{n}\right|}_{X_{n}=t}=\left\{\begin{array}{ll} s, & \text{if}\;\left|S_{n-1}\right|>\left|T_{n-1}\right|+1,\\ t, & \text{if}\;\left|S_{n-1}\right|\leq \left|T_{n-1}\right|+1. \end{array}\right.$$ (8)

The difference, *d*_*n*_ = |*S*_*n*−1_| − |*T*_*n*−1_|, in votes to *s* and *t* by the preceding casting voters of the *n*-th respondent can be regarded as a random walk on the integer steps from −2 to 2. While *d*_*n*_ is in −1, 0, or 1, the *n*-th respondent is a casting voter, and *d*_*n*+1_ increases or decreases by 1 from *d*_*n*_ depending on his/her primary choice. Once *d*_*n*_ reaches either 2 or −2, it stops changing, and all the subsequent respondents, including the *n*-th, will be non-casting voters. The magic numbers 2 and −2 of vote difference are therefore absorbing states in the sequential decision-making of respondents with equal abilities. Once one of the absorbing states is reached by a ‘random’ vote of a casting voter relying only on his/her primary choice, there will be no improvement in the collective intelligence accuracy thereafter (i.e. collective intelligence dies there, the decision of the last casting voter enters the hall of fame, and everyone thereafter stops thinking and follows it). Collective intelligence lives only until the preceding votes are too close to a call (|*d*_*n*_| ≤ 1).

The mean performance E[*π*_*n*_] = E[*π*_*n*_(*S*_*n*−1_, *T*_*n*−1_, *R*_*n*−1_, *X*_*n*_)] of the *n*-th individual can be written as:

$$\text{E}\left[\pi_{n}\right]=\frac{p^{2}+p\left(1-p\right)\left(1-2p\right){\left[2p\left(1-p\right)\right]}^{k-1}}{p^{2}+{\left(1-p\right)}^{2}}.$$ (9)

**Fig 4:**
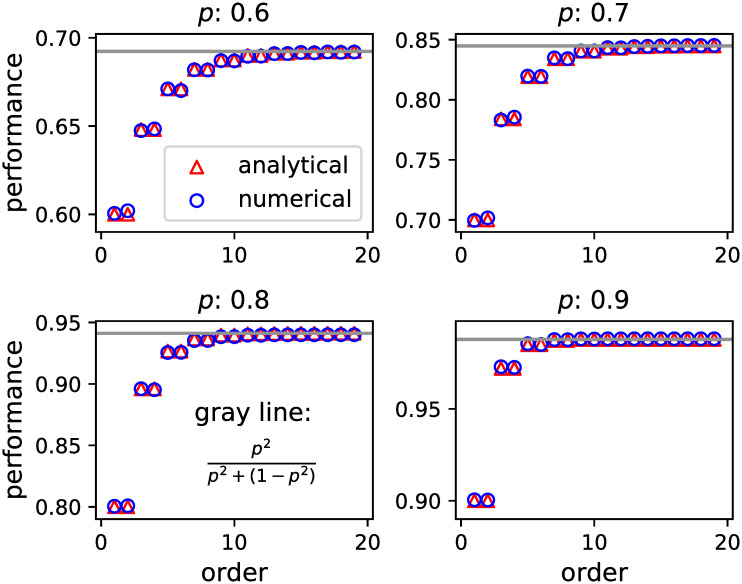
Mean performance versus order. Simulation (blue circles) and analytical (red triangles) results of mean performance E[*π*_*n*_] (ordinate) versus order *n* (abscissa) are exhibited for each individual ability *p* in each panel. The horizontal grey line shows the value of *π*_max_(*p*).

As 1/2 < *p* < 1 is assumed, *p*(1 − *p*)(1 − 2*p*) is negative, and 2*p*(1 − *p*) < 1. Therefore, *E*[*π*_*n*_] increases for every odd number *n* and approaches an upper bound *π*_max_(*p*) = *p*^2^/[*p*^2^ + (1 − *p*)^2^] in the limit of infinitely large *n*. Fig 4 shows the simulation and analytical results of E[*π*_*n*_] plotted against *n*, for each ability *p* of individuals. The analytical result was calculated using Eq (9). The simulation result was calculated based on an agent-based simulation, where each individual makes his/her primary choice, recursively updates sets *S*_*m*_, *T*_*m*_, and *R*_*m*_(*m* = 1, …, *n* − 1) using the algorithm in Table 1, and determines the optimal choice according to *d*_*n*_ using Eqs (7) and (8). The sequential decision-making was simulated for 10,000 runs, and the *n*-th individual’s performance was derived as the proportion of the number of correct answers by the *n*-th individual to the total number of runs (10, 000).

For comparison, we also considered the mean performance $\text{E}\left[\pi_{n}^{C}\right]$ in simultaneous decision-making with a majority vote among *n* individuals whose abilities are *p*. The mean performance $\text{E}\left[\pi_{n}^{C}\right]$ can be calculated as follows:

$$\text{E}\left[\pi_{n}^{C}\right]=\sum_{m=\left(n+1\right)/2}^{n}\left(\begin{array}{c} n\\ m \end{array}\right)p^{m}{\left(1-p\right)}^{n-m},$$ (10)

where *n* is odd, as generally assumed in the literature on collective intelligence [2]. It is well known that by the Condorcet jury theorem, ${\text{lim}}_{n\to \infty }\;\text{E}\left[\pi_{n}^{C}\right]=1$, provided that *p* > 1/2. We determine what number, *n*_*e*_, of voters in simultaneous decision-making is necessary to make the mean performance as large as the upper bound of the mean performance in sequential decision making:

$$\sum_{m=\left(n_{e}+1\right)/2}^{n_{e}}\left(\begin{array}{c} n_{e}\\ m \end{array}\right)p^{m}{\left(1-p\right)}^{n_{e}-m}=\frac{p^{2}}{p^{2}+{\left(1-p\right)}^{2}}=\pi_{\text{max}}\left(p\right).$$ (11)

We call *n*_*e*_ the effective number of voters for sequential decision-making. We found from Eq (11) that, irrespective of how large the total number *N* of respondents in sequential decision-making is, the effective number remains small: 3−5 when $0.5<p<\left(3+\sqrt{3}\right)/6\approx 0.79$ and 5−7 when $\left(3+\sqrt{3}\right)/6<p<1$. Therefore, individuals are apt to perform much less in sequential decision-making than in simultaneous decision-making; even if a large number of individuals participate in sequential decision-making, the majority vote of just three to five individuals would suffice if the individuals’ abilities fall between 50% and 80%, or a majority vote of just five to seven individuals would suffice if the abilities of the individuals are even higher (80–100%). In this sense, simultaneous decision-making, where independent answers are aggregated, is better than sequential decision-making.

As discussed earlier, the *n*-th individual’s primary choice is the casting vote only when he/she observes |*d*_*n*_| = ||*S*_*n*−1_| − |*T*_*n*−1_|| ≤ 1 in the answers of her antecedents. Once an individual observes |*d*_*n*_| = 2 in the answers of her antecedents, he/she and all subsequent persons will be non-casting voters. Therefore, the number of antecedents assigned to either *S*_*n*−1_ or *T*_*n*−1_ should be much lower than that assigned to *R*_*n*−1_ at the *n*-th individual’s decision-making for large *n*. The *n* th individual cannot use the information on the primary choices of antecedents in *R*_*n*−1_. Thus, he/she cannot have a high performance that could be realised when answers by his/her antecedents are independent of each other.

#### One person having a higher ability than others

The above analyses pertain to the case in which every respondent has the same ability. Here, we explore the case where one individual, called an *expert*, has a higher ability *q* than others having abilities *p*(*q* > *p*). The order decided by the expert is denoted by *n*, and the mean performance is denoted by *E*[*π*_*n*,*q*_]. Here, we investigated the mean performance of the expert considering two contrasting cases for the awareness of the expert regarding his/her ability.

First, we considered a case wherein the expert knows his/her ability *q*. The optimal behaviour is shown to be identical to that of the *n*-th respondent with the same ability *p* as others if *q* < *p*^2^/(*p*^2^ + (1 − *p*)^2^) = *π*_max_(*p*), i.e., he/she should respond with his/her primary choice when |*d*_*n*_| ≤ 1, while he/she should follow the majority vote of the preceding voters when |*d*_*n*_| = 2. Contrarily, if *q* > *π*_max_(*p*), the expert should always respond with his/her primary choice regardless of the preceding casting vote distribution. The mean performance of the expert when he/she is the *n*-th respondent is

$$\text{E}\left[\pi_{n,q}\right]=\left\{\begin{array}{ll} \frac{p^{2}}{p^{2}+{\left(1-p\right)}^{2}}-\left(\frac{p^{2}}{p^{2}+{\left(1-p\right)}^{2}}-q\right){\left[2p\left(1-p\right)\right]}^{k-1}, & \text{if}\;q<\pi_{\text{max}}\left(p\right),\\ q, & \text{if}\;\text{otherwise} \end{array}\right.$$ (12)

where *k* = (*n* + 1)/2 if *n* is odd, and *k* = *n*/2 if *n* is even. When *q* < *π*_max_(*p*), the later the expert makes his/her decision, the greater the performance, which approaches *π*_max_(*p*) for an infinitely late decision. By contrast, when *q* ≥ *π*_max_(*p*), the order of the decision does not affect the performance at all and is equal to *q*.

Second, we considered the case wherein the expert is not aware of his/her superiority over others in terms of ability. In this case, the expert responds with his/her primary choice when |*d*_*n*_| ≤ 1 and follows the majority vote in the preceding votes when |*d*_*n*_| = 2. Therefore, the mean performance of the expert is

$$E\left[\pi_{n,q}\right]=\frac{p^{2}}{p^{2}+{\left(1-p\right)}^{2}}-\left(\frac{p^{2}}{p^{2}+{\left(1-p\right)}^{2}}-q\right){\left[2p\left(1-p\right)\right]}^{k-1}.$$ (13)

**Fig 5:**
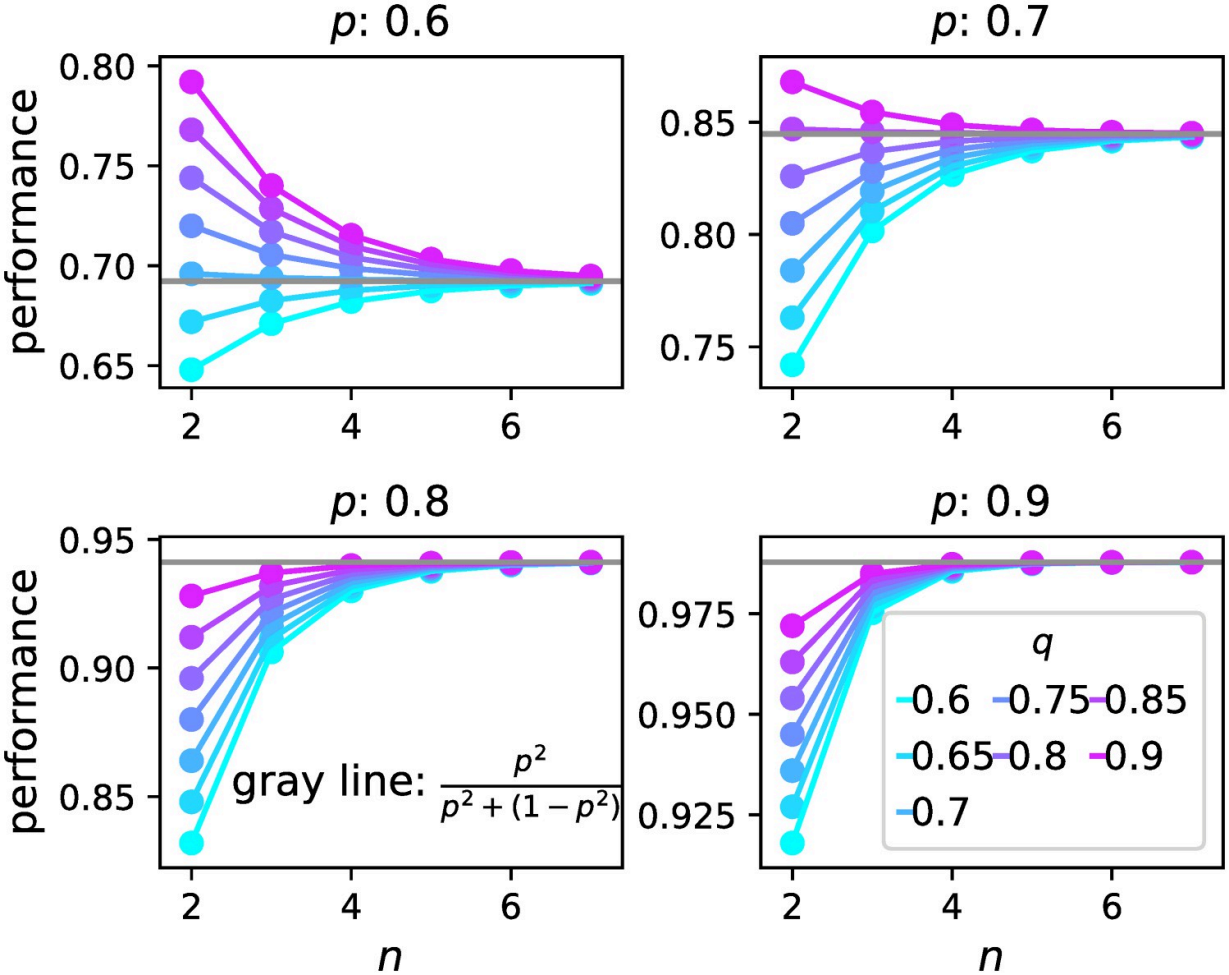
Mean performance E[*π*_*n*,*q*_] of the expert with ability *q* when the expert believes that his/her ability is *p*. Each panel exhibits E[*π*_*n*,*q*_] as the other individuals have the ability *p* = 0.6, 0.7, 0.8 or 0.9. The colour of the plots stands for the value of *q*. The horizontal line (grey) shows the value of *π*_max_(*p*).

Interestingly, while the mean performance of the expert increases with the decision order *n* when *q* < *π*_max_ (*p*) as in the case where he/she is aware of his/her superiority in terms of ability, it decreases with the decision order *n* if *q* > *π*_max_ (*p*) (Fig 5). In other words, a person who is exceptionally gifted but is not aware of it should answer earlier to get better performance, although he/she should answer later if his/her ability is not sufficiently higher than others.

## Discussion

We investigated the sequential decision-making of individuals who attempt to maximise their individual accuracies in solving a binary choice problem by observing the answers of their antecedents. We call the primary choice of an individual the casting vote if his/her optimum answer is to choose his/her primary choice, regardless of the antecedents’ answers. We also suggested an algorithm for finding casting voters among antecedents. By considering the abilities and answer distribution of only casting voters, one can calculate which of the two alternatives has a higher likelihood of being correct. By applying this theory to the optimal behaviour for sequential decision-making involving three individuals, we observed a counterintuitive phenomenon where the mean performance of the third respondent worsened when the first respondent had a higher ability than the second. We also investigated the sequential decision-making of an arbitrary number of individuals with the same ability. The results revealed that individuals could improve their mean performance by answering questions as late as possible. We also found that for a large number of respondents, their mean performance is only comparable to that of simultaneous decision-making of at most five respondents. Therefore, when an individual with sufficiently high ability joins the sequential decision-making of respondents with homogeneous ability, he/she is never motivated to consider others’ answers.

Casting voters, as a result of their optimal behaviours, give the later answering respondents information on their primary choices, which help later respondents to improve their mean performance. In one’s decision-making, the likelihood of an alternative being correct can be derived from the opinion distribution of the primary choices of antecedent casting voters. These primary choices are stochastically determined and unobservable by others. However, individuals can always correctly guess the primary choices of casting voters because answers by casting voters are always as valuable as their primary choices.

Our analysis explained why the opinion correlation generated by non-casting voters negatively affects the mean performance of later respondents. We revealed that in the sequential decision-making involving three individuals, the performance of the third individual was lower when the ability of the first was greater than that of the second. In this case, the second individual was a non-casting voter and only followed his/her antecedent to generate an opinion correlation between them. Our study also showed that, in the sequential decision-making of arbitrary number of individuals with the same ability, respondents become non-casting voters and give correlated answers once the difference in antecedents’ votes to two options reaches 2. Accordingly, the mean performance of respondents never exceeds the accuracy of simultaneous decision-making involving only seven individuals. Previous studies have also discussed that, if individuals incorporate social information to improve their decision accuracy, their opinions become correlated to each other and their diversity eventually decreases, leading to a deterioration of collective intelligence [2, 23, 25, 38]. In our model of sequential decision-making, the causality between the correlation among answers and deterioration of the decision accuracy of later respondents was clear, indicating that some of the respondents did not open their primary choices to the public by following their antecedents, which prevented later respondents from referring to them.

Respondents’ optimal decision-making significantly depends on the decision order of individuals with the heterogeneous abilities and can be sometimes counter intuitive. One may think that the *n*-th respondent should decide by him/herself without considering antecedents’ answers if he/she has a higher ability than all the antecedents. However, even when he/she has the highest ability, he/she should be a non-casting voter and use the collective intelligence effect generated by antecedents if the difference in total weights voted to two options by preceding casting voters is larger than his/her weight. Our results also suggest that all respondents other than the first become non-casting voters if the first respondent has the highest ability of all. This can be shown similarly to the calculation of optimal behaviour in the case of three persons with arbitrary abilities (the section ‘Specific cases’); the *n*-th respondent becomes a non-casting voter if *p*_1_ > *p*_*n*_ and from the second to the (*n* − 1)-th respondents are non-casting voters. In this case, all respondents answer the same option as the first. Note that, while an information cascade, when the first respondent answers incorrectly all the other individuals answer incorrectly, can occur, being a non-casting voter is the optimal strategy for each to obtain the highest mean conditional performance.

We predicted that if individuals are allowed to decide when to answer by themselves in collective decision-making, those who have low abilities can face a dilemma: each wants others with low abilities to answer quickly but they themselves want to answer late. How the decision order of individuals with different levels of information is self-organised has been an attractive question in the literature on collective decision-making [30, 39–41]. We revealed that a gifted individual with sufficiently higher ability than all others with the same ability can perform well whenever he/she answers. By contrast, individuals with lower abilities can increase their performance by waiting to consider several antecedents’ answers for as long as possible. Therefore, the motivation to wait for others’ opinions is much higher for individuals with low abilities. However, at the same time, a low-ability individual can achieve a higher performance when other individuals with low abilities answer the earliest because the correlation among the antecedents’ answers is suppressed in such a decision order.

In our sequential decision-making model, we showed that the procedure of optimal decision-making based on the antecedents’ decision is simple and robust to small errors in inferring others’ abilities. In this study, we assumed sequential decision-making where each respondent attempts to maximise his/her conditional performance knowing that each of the antecedents behaved optimally. Intuitively, such optimal decision-making requires information on the inner decision process or unobservable primary choices of all antecedents. We showed that each can reduce the computational cost of his/her optimal behaviour by ignoring the answers of non-casting voters. Furthermore, he/she can guess the primary choices of casting voters without any uncertainty. His/her optimal behaviour is also determined without any uncertainty by comparing the summation of the log-odds ratios of the casting voters’ abilities, which are well-known optimum weights in simultaneous weighted majority vote. One factor which simplifies the optimal decision calculation is the assumption that individuals know the abilities of their antecedents and themselves. While the precise estimation of others’ abilities for a given problem is difficult in practice, people can estimate them through repeated social interaction [42] or records of others’ decision-making. For example, some platforms of future forecasts or crowdsourcing record the guesses or performance of agents for reference [6, 9]. Moreover, optimal decision-making in this study is determined by evaluating inequalities rather than equalities (Eqs (2) and (3)). Regarding the condition in Eq (2), for example, a respondent should consider two regions determined by whether the difference in total weights voted to two options by preceding casting voters is larger than the weight of the focal respondent. He/she then determines the region to which the point of optimum weights $\left(r_{1}^{*},\ldots ,r_{n}^{*}\right)$ falls, in his/her optimal behaviour. Here, even when the estimated values of optimum weights slightly deviate from the true values, the deviated point should still fall into the same region in many cases. Therefore, the optimal behaviour is robust against small errors in the estimation of abilities. In contrast, a large deviation of ability estimation from the true value can hinder an individual’s optimal decision-making. Such deviation can occur in practice for various reasons; for example, some antecedents may deceive others. Similarly, an individual having a strong social impact may receive more weight than expected with his/her actual ability. It should be interesting to study how the failure in ability estimation or weighing affects the decision accuracy of each respondent.

In future work, we would like to empirically investigate whether humans can judge a preceding individual as a casting voter and whether they tend to ignore the opinions of non-casting voters, as our study of optimal decision-making suggests. Empirical studies have also revealed that when subjects cannot estimate others’ abilities because of the absence of repeated social interaction, they weigh others’ opinions based on expressed confidence [15, 43–45], which is motivated by the confidence-accuracy relationship. A possible question is whether individuals can weigh their antecedents and themself nearly optimally based on their confidence in sequential decision-making.

## Supporting information

S1 Text Supplementary information of casting votes of antecedents play a key role in successful sequential decision-making. In this paper, we presented a detailed calculation for determining the optimal behaviour and performance of individuals in simultaneous decision-making and sequential decision-making.
(PDF)

## References

[1] Deutsch M, Gerard HB. A study of normative and informational social influences upon individual judgment. J Abnorm Soc Psychol. 1955;51: 629. 10.1037/h0046408 13286010

[2] Ladha KK. The Condorcet jury theorem, free speech, and correlated votes. Am J Polit Sci. 1992; 617–634. doi: 10.2307/2111584

[3] Francis G. Vox Populi. Nature. 1907;75: 450–51.

[4] Krause J, Ruxton GD, Krause S. Swarm intelligence in animals and humans. Trends Ecol Evol. 2010;25: 28–34. doi: 10.1016/j.tree.2009.06.016 19735961

[5] Woolley AW, Chabris CF, Pentland A, Hashmi N, Malone TW. Evidence for a collective intelligence factor in the performance of human groups. science. 2010;330: 686–688. doi: 10.1126/science.1193147 20929725

[6] Budescu DV, Chen E. Identifying expertise to extract the wisdom of crowds. Manag Sci. 2015;61: 267–280. doi: 10.1287/mnsc.2014.1909

[7] Arrow KJ, Forsythe R, Gorham M, Hahn R, Hanson R, Ledyard JO, et al. The Promise of Prediction Markets. Science. 2008;320: 877–878. doi: 10.1126/science.1157679 18487176

[8] Pfeiffer T, Almenberg J. Prediction markets and their potential role in biomedical research–a review. Biosystems. 2010;102: 71–76. doi: 10.1016/j.biosystems.2010.09.005 20837097

[9] Mitry D, Peto T, Hayat S, Blows P, Morgan J, Khaw K-T, et al. Crowdsourcing as a screening tool to detect clinical features of glaucomatous optic neuropathy from digital photography. PloS One. 2015;10: e0117401. doi: 10.1371/journal.pone.0117401 25692287 PMC4334897

[10] Aspinall W. A route to more tractable expert advice. Nature. 2010;463: 294–295. doi: 10.1038/463294a 20090733

[11] Klein N, Epley N. Group discussion improves lie detection. Proc Natl Acad Sci U S A. 2015;112: 7460–7465. 26015581 10.1073/pnas.1504048112 PMC4475962

[12] Kurvers RHJM, Herzog SM, Hertwig R, Krause J, Carney PA, Bogart A, et al. Boosting medical diagnostics by pooling independent judgments. Proc Natl Acad Sci U S A. 2016;113: 8777–8782. doi: 10.1073/pnas.1601827113 27432950 PMC4978286

[13] Juni MZ, Eckstein MP. The wisdom of crowds for visual search. Proc Natl Acad Sci. 2017;114: E4306–E4315. doi: 10.1073/pnas.1610732114 28490500 PMC5448189

[14] Zhou Z-H. Ensemble methods: foundations and algorithms. Cambridge: Chapman and Hall/CRC; 2019.

[15] Bahrami B, Olsen K, Latham PE, Roepstorff A, Rees G, Frith CD. Optimally interacting minds. Science. 2010;329: 1081–1085. doi: 10.1126/science.1185718 20798320 PMC3371582

[16] Nitzan S, Paroush J. Optimal decision rules in uncertain dichotomous choice situations. Int Econ Rev. 1982; 289–297. doi: 10.2307/2526438

[17] Nitzan S. Collective preference and choice. Cambridge: Cambridge University Press; 2009.

[18] Shapley L, Grofman B. Optimizing group judgmental accuracy in the presence of interdependencies. Public Choice. 1984;43: 329–343. doi: 10.1007/BF00118940

[19] Baharad E, Goldberger J, Koppel M, Nitzan S. Beyond Condorcet: Optimal aggregation rules using voting records. Theory Decis. 2012;72: 113–130. doi: 10.1007/s11238-010-9240-5

[20] Grofman B, Owen G, Feld SL. Thirteen theorems in search of the truth. Theory Decis. 1983;15: 261–278. doi: 10.1007/BF00125672

[21] Marshall JA, Brown G, Radford AN. Individual confidence-weighting and group decision-making. Trends Ecol Evol. 2017;32: 636–645. doi: 10.1016/j.tree.2017.06.004 28739079

[22] Lorenz J, Rauhut H, Schweitzer F, Helbing D. How social influence can undermine the wisdom of crowd effect. Proc Natl Acad Sci U S A. 2011;108: 9020–9025. doi: 10.1073/pnas.1008636108 21576485 PMC3107299

[23] Kao AB, Couzin ID. Decision accuracy in complex environments is often maximized by small group sizes. Proc R Soc B Biol Sci. 2014;281: 20133305. doi: 10.1098/rspb.2013.3305 24759858 PMC4043084

[24] Ito MI, Ohtsuki H, Sasaki A. Emergence of opinion leaders in reference networks. PloS One. 2018;13: e0193983. doi: 10.1371/journal.pone.0193983 29579053 PMC5868794

[25] Madirolas G, de Polavieja GG. Improving collective estimations using resistance to social influence. PLoS Comput Biol. 2015;11: e1004594. doi: 10.1371/journal.pcbi.1004594 26565619 PMC4643903

[26] Mori S, Hisakado M, Takahashi T. Phase transition to a two-peak phase in an information-cascade voting experiment. Phys Rev E. 2012;86: 026109. doi: 10.1103/PhysRevE.86.026109 23005827

[27] Pérez-Escudero A, de Polavieja GG. Collective Animal Behavior from Bayesian Estimation and Probability Matching. PLoS Comput Biol. 2011;7: e1002282. doi: 10.1371/journal.pcbi.1002282 22125487 PMC3219619

[28] Arganda S, Pérez-Escudero A, de Polavieja GG. A common rule for decision making in animal collectives across species. Proc Natl Acad Sci U S A. 2012;109: 20508–20513. doi: 10.1073/pnas.1210664109 23197836 PMC3528575

[29] Eguíluz VM, Masuda N, Fernández-Gracia J. Bayesian decision making in human collectives with binary choices. PLoS One. 2015;10: e0121332. doi: 10.1371/journal.pone.0121332 25867176 PMC4395091

[30] Tump AN, Pleskac TJ, Kurvers RHJM. Wise or mad crowds? The cognitive mechanisms underlying information cascades. Sci Adv. 2020;6: eabb0266. doi: 10.1126/sciadv.abb0266 32832634 PMC7439644

[31] Bikhchandani S, Hirshleifer D, Welch I. A theory of fads, fashion, custom, and cultural change as informational cascades. J Polit Econ. 1992;100: 992–1026.

[32] King AJ, Cheng L, Starke SD, Myatt JP. Is the true ‘wisdom of the crowd’ to copy successful individuals? Biol Lett. 2012;8: 197–200. doi: 10.1098/rsbl.2011.0795 21920956 PMC3297389

[33] Khalvati K, Mirbagheri S, Park SA, Dreher J-C, Rao RP. A Bayesian theory of conformity in collective decision making. Adv Neural Inf Process Syst. 2019;32.

[34] Khalvati K, Park SA, Mirbagheri S, Philippe R, Sestito M, Dreher J-C, et al. Modeling other minds: Bayesian inference explains human choices in group decision-making. Sci Adv. 2019;5: eaax8783. doi: 10.1126/sciadv.aax8783 31807706 PMC6881156

[35] Devaine M, Hollard G, Daunizeau J. Theory of mind: did evolution fool us? PloS One. 2014;9: e87619. doi: 10.1371/journal.pone.0087619 24505296 PMC3914827

[36] Jara-Ettinger J. Theory of mind as inverse reinforcement learning. Curr Opin Behav Sci. 2019;29: 105–110. doi: 10.1016/j.cobeha.2019.04.010

[37] Vélez N, Gweon H. Integrating incomplete information with imperfect advice. Top Cogn Sci. 2019;11: 299–315. doi: 10.1111/tops.12388 30414253

[38] Mann RP, Helbing D. Optimal incentives for collective intelligence. Proc Natl Acad Sci U S A. 2017;114: 5077–5082. doi: 10.1073/pnas.1618722114 28461491 PMC5441831

[39] Kurvers RHJM, Wolf M, Naguib M, Krause J. Self-organized flexible leadership promotes collective intelligence in human groups. R Soc Open Sci. 2015;2: 150222. doi: 10.1098/rsos.150222 27019718 PMC4807439

[40] Boos M, Pritz J, Lange S, Belz M. Leadership in moving human groups. PLoS Comput Biol. 2014;10: e1003541. doi: 10.1371/journal.pcbi.1003541 24699264 PMC3974633

[41] Guttman I. The timing of analysts’ earnings forecasts. Account Rev. 2010;85: 513–545. doi: 10.2308/accr.2010.85.2.513

[42] Behrens TE, Hunt LT, Woolrich MW, Rushworth MF. Associative learning of social value. Nature. 2008;456: 245–249. doi: 10.1038/nature07538 19005555 PMC2605577

[43] Koriat A. When are two heads better than one and why? Science. 2012;336: 360–362. doi: 10.1126/science.1216549 22517862

[44] Bang D, Aitchison L, Moran R, Herce Castanon S, Rafiee B, Mahmoodi A, et al. Confidence matching in group decision-making. Nat Hum Behav. 2017;1: 1–7. doi: 10.1038/s41562-017-0117

[45] Pescetelli N, Yeung N. The role of decision confidence in advice-taking and trust formation. J Exp Psychol Gen. 2021;150: 507. doi: 10.1037/xge0000960 33001684

