# Supplementary material for: Casting votes of antecedents play a key role in successful sequential decision-making
Source: PLoS One. 2023 Feb 24;18(2):e0282062. doi: 10.1371/journal.pone.0282062 (PMC9955594; doi:10.1371/journal.pone.0282062)
Supplement: S1 Text — In this paper, we presented a detailed calculation for determining the optimal behaviour and performance of individuals in simultaneous decision-making and sequential decision-making. (PDF) [file pone.0282062.s001.pdf]

Supplementary information of Casting votes of antecedents play a key role in successful sequential decision-making

Mariko I. Ito and Akira Sasaki

In this paper, we presented a detailed calculation for determining the optimal behaviour and performance of individuals in simultaneous decision-making (Section S.1) and sequential decision-making (Section S.2).

S.1 Simultaneous decision-making

In this section, we assumed simultaneous decision-making by multiple individuals, where each individual votes independently, and their votes are aggregated simultaneously by a weighted majority rule, as shown in Section 2.2 of the main text. The optimum weights for each individual in simultaneous decision-making have been discussed in previous studies [1–3]. Here, we revisit the calculation of optimum weights in previous studies.

S.1.1 General case

Optimum weights to maximize conditional performance

We assume N individuals, individuals 1, 2,..., N, and consider two sets S and T. Set S consists of the indices of individuals who voted s, and T consists of those who voted t. Therefore, $S \sqcup T = \{1, 2, ..., N\}$. The opinion distribution, where individuals are separated into S and T, is denoted by (S, T). Let w_n be the weight of the vote of individual n in the weighted majority vote ($\sum_{n=1}^{N} w_n = 1$). The outcome of the weighted majority vote is s if $W(S) = \sum_{n \in S} w_n > 0.5$ and t if $W(S) < 0.5$; that is, $W(T) > 0.5$. To simplify the discussion, we do not consider weights $\{w_n\}$ that satisfy $W(S) = W(T) = 0.5$. Let p_n be the ability of individual n, which is the probability of voting for the correct alternative.

We first assume that neither S nor T is empty. The probability of obtaining the opinion distribution (S, T) of individuals when alternative s is correct is $L(S) := P(S)Q(T)$, where $P(X) = \prod_{n \in X} p_n$ and $Q(X) = \prod_{n \in X}(1 - p_n)$. In other words, $L(S)$ is the likelihood that alternative s is correct given the opinion distribution (S, T). By contrast, the likelihood that alternative t is correct, given the votes, is written as $L(T) = P(T)Q(S)$.

Therefore, given the opinion distribution (S, T), the conditional probability of s being correct is calculated as

$$\text{Prob}[s \text{ is correct } |(S,T)] = \frac{\text{Prob}[s \text{ is correct }, (S,T)]}{\text{Prob}[(S,T)]} = \frac{P(S)Q(T)}{Z_{(S,T)}} = \frac{L(S)}{Z_{(S,T)}}, \quad \text{(S.1)}$$

where $Z_{(S,T)} = \text{Prob}[(S,T)] = \text{Prob}[s \text{ is correct}, (S,T)] + \text{Prob}[t \text{ is correct}, (S,T)] = P(S)Q(T) + Q(S)P(T) = L(S) + L(T)$. Similarly, the conditional probability of t being correct given the opinion distribution (S,T) is written as

$$\text{Prob}[t \text{ is correct } |(S,T)] = \frac{L(T)}{Z_{(S,T)}}. \tag{S.2}$$

To simplify the discussion, we do not consider sets of abilities that satisfy $L(S) = L(T)$. Given opinion distribution (S,T), let us call the conditional probability of the outcome of a weighted majority vote correct *conditional performance*. Therefore, the conditional performance given (S,T) is $L(S)/Z_{(S,T)}$ when the outcome of the weighted majority vote is s, and it is $L(T)/Z_{(S,T)}$ when the outcome is t.

Let us consider a decision procedure in which the outcome of the weighted majority vote is stochastically determined as s with probability $\alpha(< 1)$, t otherwise, given opinion distribution (S,T). In this case, the conditional performance becomes $\left[\alpha L(S) + (1-\alpha)L(T)\right]/Z_{S,T}$, which is less than $L(S)/Z_{S,T}$ if $L(S) > L(T)$ and less than $L(T)/Z_{S,T}$ if $L(S) < L(T)$. Therefore, when $L(S) > L(T)$, the outcome of the weighted majority vote should be s with probability 1 to maximise conditional performance, which results in $L(S)/Z_{S,T}$. Choice s can be the outcome of the weighted majority vote if and only if the weights satisfy

$$W(S) > W(T), \text{ i.e., } W(S) > 0.5. \tag{S.3}$$

Note that the condition $L(S) > L(T)$ means that the likelihood that s is correct is greater than the likelihood that t is correct and is equivalent to

$$\sum_{n \in S} r_n^* > \sum_{n \in T} r_n^*, \tag{S.4}$$

where

$$r_n^* := \log \left[\frac{p_n}{1 - p_n} \right]. \tag{S.5}$$

In summary, when $\sum_{n \in S} r_n^* > \sum_{n \in T} r_n^*$, the weights should always satisfy $W(S) > W(T)$ to ensure that s is the outcome of the weighted majority vote with a probability of 1. A similar discussion can also be applied to the case of $L(S) < L(T)$, i.e., $\sum_{n \in S} r_n^* < \sum_{n \in T} r_n^*$.

Thus, given the opinion distribution (S, T) of votes, the condition that weights should be satisfied to maximise the conditional performance of the weighted majority vote can be summarised as follows. Weights should satisfy the following:

A) $W(S) > W(T)$ when the likelihood of s being correct is greater than that of t, i.e. $\sum_{n \in S} r_n^* > \sum_{n \in T} r_n^*$, and

B) $W(S) < W(T)$ when the likelihood of t being correct is greater than that of s, i.e. $\sum_{n \in S} r_n^* < \sum_{n \in T} r_n^*$.

When all individuals' votes are the same, that is, either S or T is empty, the outcome should be the alternative chosen by them because $\prod_{n=1}^{N} p_n > \prod_{n=1}^{N} (1 - p_n)$ is always satisfied as $p_n > 0.5$, and the likelihood that their consensus is correct is greater than

that of the opposite alternative. In fact, their consensus always becomes the outcome of a weighted majority vote, because $\sum_{n=1}^{N} w_n = 1 > 0.5$. Therefore, by defining $P(X) = Q(X) = 1$ for an empty set X, we can also determine the optimum weights to maximise the conditional performance given (S, T) where either S or T is empty according to A) and B).

Based on the discussion thus far, the conditional performance given (S, T), which is maximised by assigning weights as the rules of A) and B), can be written as

$$\max\left[\frac{L(S)}{Z_{(S,T)}}, \frac{L(T)}{Z_{(S,T)}}\right]. \tag{S.6}$$

Geometrical interpretation of condition for optimum weights

Here, we provide a geometrical interpretation of the condition of weights for maximising the conditional performance of the weighted majority vote given the opinion distribution (S, T). We consider $(N-1)$-simplex as

$$\left\{\mathbf{w} = (w_1, w_2, ..., w_N);\ \sum_{n=1}^{N} w_n = 1\right\}, \tag{S.7}$$

and the hyperplane is determined by the equation $W(S) = W(T) = 0.5$. Given the opinion distribution (S, T), by comparing which is greater between $\sum_{n \in S} r_n^*$ and $\sum_{n \in T} r_n^*$, we can determine the domain $D_{(S,T)}$ of weights that maximise the conditional performance according to the rules of A) and B). $D_{(S,T)}$ is one of the two domains in an $(N-1)$ simplex

divided by the hyperplane determined by $W(S) = W(T)$:

$$D_{(S,T)} = \begin{cases} \{\mathbf{w};\ W(S) > W(T), \sum_{n=1}^{N} w_n = 1\} & \text{if } \sum_{n\in S} r_n^* > \sum_{n\in T} r_n^*, \\ \{\mathbf{w};\ W(S) < W(T), \sum_{n=1}^{N} w_n = 1\} & \text{if } \sum_{n\in S} r_n^* < \sum_{n\in T} r_n^*. \end{cases} \tag{S.8}$$

Specifically, $D_{(S,T)} = \{\mathbf{w};\ \sum_{n=1}^{N} w_n = 1\}$ (the entire region of $(N-1)$)-simplex) when S or T is empty, that is, when all individuals vote for the same alternative.

Given individual abilities, the domain of weights that maximise conditional performance for any opinion distribution (S,T), $\bigcap_{(S,T)} D_{S,T}$, is not empty because $\bigcap_{(S,T)} D_{S,T}$ includes the point of $\mathbf{r}^*/Z = (r_1^*/Z, r_2^*/Z, ..., r_N^*/Z)$, where $Z = \sum_{n=1}^{N} r_n^*$. The weight $\mathbf{r}^*/Z$ satisfies conditions A) and B) for any separation (S,T). Indeed, it is well known that the relative log-odds ratio of an individual's ability gives the optimum weight to maximise the accuracy of the results of the weighted majority vote [1, 2, 4]. As $D_{S,T}$ is an open set for each (S,T), the intersection $\bigcap_{(S,T)} D_{S,T}$ is not a point but an open set, that is, points other than $\mathbf{r}^*/Z$ are in $\bigcap_{(S,T)} D_{S,T}$. Shapley and Grofman (1984) also noted that the optimum weight of an individual with a certain ability is not unique and can take values other than the relative log-odds ratio [2]. However, to the best of our knowledge, no study has geometrically summarised the region of optimum weights to maximise the conditional performance for any opinion distribution (S,T) given individuals' abilities, as shown in this section.

The weights in the domain $\bigcap_{(S,T)} D_{S,T}$ including $\mathbf{r}^*/Z$ also maximise the mean of the

conditional performance over all possible opinion distributions (S, T), which is called the *mean performance*. The mean performance of the weighted majority vote with weights in $\bigcap_{(S,T)} D_{S,T}$ can be calculated as follows:

$$\sum_{(S,T)} \text{Prob}[(S,T)] \max\left[\frac{L(S)}{Z_{(S,T)}}, \frac{L(T)}{Z_{(S,T)}}\right]$$
$$= \sum_{(S,T)} \max\left[L(S), L(T)\right]. \tag{S.9}$$

It is also shown by Eq. (S.9) that the weights should be in $\bigcap_{(S,T)} D_{S,T}$ to maximise mean performance.

Expert rule

In the case in which abilities satisfy $r_n^* > \prod_{m \neq n} r_m^*$, the likelihood of the alternative chosen by individual n being correct is always greater than that of the opposite one for any opinion distribution. Therefore, for any opinion distribution, the sum of the weights of individuals whose votes are the same as that of individual n is always greater than 0.5. Thus, $\bigcap_{(S,T)} D_{S,T}$ is $\left\{\mathbf{w}; w_n > 0.5, \sum_{n=1}^{N} w_n = 1\right\}$. The condition $w_n > 0.5$ means that the outcome of the weighted majority vote is always the same as the vote by individual n. The resulting mean performance of the weighted majority vote is then only the ability p_n of individual n. Let us call the collective decision-making method *expert rule* governed by individual n when the outcome is always the same as the vote of only a single individual, regardless of separation (S, T).

S.1.2 Two individuals

We apply the calculation shown in Section S.1.1 to the case of simultaneous decision-making by two individuals.

When two individuals' votes are the same, the outcome of the weighted majority vote should be the alternative chosen by them to maximise the conditional performance, given the distribution of votes. In this case, where S or T is empty, the domain of optimum weights $D_{S,T}$ is $(N-1)$-simplex.

If their primary choices are different from each other, according to rules A) and B) in the previous section, $w_1 > w_2$ should be satisfied, and $D_{S,T} = \{\mathbf{w}; w_1 > w_2, w_1 + w_2 = 1\}$ to maximise the conditional performance, given two different votes when $r_1^* > r_2^*$ (i.e. $p_1 > p_2$). Similarly, $w_1 < w_2$ should be satisfied and $D_{S,T} = \{\mathbf{w}; w_1 < w_2, w_1 + w_2 = 1\}$ when $r_1^* < r_2^*$ (i.e. $p_1 < p_2$).

Therefore, in the case of two individuals, the domain of weights $\bigcap_{(S,T)} D_{S,T}$ that maximises the mean performance and the mean performance on such optimum weights is summarised as follows:

- If $p_1 > p_2$, $\bigcap_{(S,T)} D_{S,T} = \{\mathbf{w}; w_1 > w_2, w_1 + w_2 = 1\}$. The mean performance is $p_1 p_2 + p_1(1-p_2) = p_1$.

- If $p_1 < p_2$, $\bigcap_{(S,T)} D_{S,T} = \{\mathbf{w}; w_1 > w_2, w_1 + w_2 = 1\}$. The mean performance is $p_1 p_2 + (1-p_1)p_2 = p_2$.

The mean performance is calculated using Eq. (S.9). The first case can be interpreted as

follows: When the ability of individual 1 is greater than that of individual 2, the weights should satisfy $w_1 > 0.5$ to maximise the mean performance. Therefore, the expert rule governed by individual 1 is optimal when $p_1 > p_2$. Similarly, in the second case, the expert rule governed by individual 2 is optimal when the ability of individual 2 is greater than that of individual 1.

S.1.3 Three individuals

We apply the calculation of optimum weights shown in Section S.1.1 for simultaneous decision making by three individuals. In this case, the methods for separating their votes are either $\{1\}$ versus $\{2, 3\}$, $\{2\}$ versus $\{1, 3\}$, $\{3\}$ versus $\{1, 2\}$, or $\{1, 2, 3\}$ versus ϕ, where ϕ denotes an empty set.

When all individuals' votes are the same, the outcome of the weighted majority vote should be the alternative chosen by them to maximise the conditional performance, given this opinion distribution. In the case where the primary choices are separated as $\{i\}$ versus $\{j, k\}$, weights should be assigned to satisfy $w_i > w_j + w_k$ when $r_i^* > r_j^* + r_k^*$ and satisfy $w_i < w_j + w_k$ when $r_i^* < r_j^* + r_k^*$ to maximise the conditional performance $(i, j, k \in \{1, 2, 3\}, i \neq j \neq k)$.

Given the abilities of the three individuals, combinations of inequalities between r_i^* and $r_j^* + r_k^*$ $(i, j, k \in \{1, 2, 3\},\ i \neq j \neq k)$ that can be realised simultaneously are

1. $r_1^* > r_2^* + r_3^*$, $r_2^* < r_1^* + r_3^*$ and $r_3^* < r_1^* + r_2^*$,

2. $r_1^* < r_2^* + r_3^*$, $r_2^* > r_1^* + r_3^*$ and $r_3^* < r_1^* + r_2^*$,

3. $r_1^* < r_2^* + r_3^*$, $r_2^* < r_1^* + r_3^*$ and $r_3^* > r_1^* + r_2^*$ or

4. $r_1^* < r_2^* + r_3^*$, $r_2^* < r_1^* + r_3^*$ and $r_3^* < r_1^* + r_2^*$.

Note that when $r_i^* > r_j^* + r_k^*$ is satisfied, neither $r_j^* > r_i^* + r_k^*$ nor $r_k^* > r_i^* + r_j^*$ is possible because p_i is very large compared with p_j and p_k.

For each of the above cases, the condition of weights for maximising the mean performance and the mean performance on such optimum weights are summarised as follows:

1. $w_1 > w_2 + w_3$, $w_2 < w_1 + w_3$ and $w_3 < w_1 + w_2$ should be satisfied. Therefore $\bigcap_{(S,T)} D_{S,T} = \left\{\mathbf{w}; w_1 > w_2 + w_3, \sum_{n=1}^{3} w_n = 1\right\}$. The mean performance is $p_1p_2p_3 + p_1(1-p_2)(1-p_3) + p_1(1-p_2)p_3 + p_1p_2(1-p_3) = p_1$,

2. $w_1 < w_2 + w_3$, $w_2 > w_1 + w_3$ and $w_3 < w_1 + w_2$ should be satisfied. Therefore $\bigcap_{(S,T)} D_{S,T} = \left\{\mathbf{w}; w_2 > w_1 + w_3, \sum_{n=1}^{3} w_n = 1\right\}$. The mean performance is $p_1p_2p_3 + (1-p_1)p_2p_3 + (1-p_1)p_2(1-p_3) + p_1p_2(1-p_3) = p_2$,

3. $w_1 < w_2 + w_3$, $w_2 < w_1 + w_3$ and $w_3 > w_1 + w_2$ should be satisfied. Therefore $\bigcap_{(S,T)} D_{S,T} = \left\{\mathbf{w}; w_3 > w_1 + w_2, \sum_{n=1}^{3} w_n = 1\right\}$. The mean performance is $p_1p_2p_3 + (1-p_1)p_2p_3 + p_1(1-p_2)p_3 + (1-p_1)(1-p_2)p_3 = p_3$ and

4. $w_1 < w_2 + w_3$, $w_2 < w_1 + w_3$ and $w_3 < w_1 + w_2$ should be satisfied. Therefore $\bigcap_{(S,T)} D_{S,T} = \left\{\mathbf{w}; w_1 < w_2 + w_3, w_2 < w_1 + w_3, w_3 < w_1 + w_2, \sum_{n=1}^{3} w_n = 1\right\}$. The mean performance is $p_1p_2p_3 + (1-p_1)p_2p_3 + p_1(1-p_2)p_3 + p_1p_2(1-p_3) =: M$,

where the mean performance is calculated according to Eq. (S.9). The first case can be interpreted as follows. Individual 1 is so excellent that $r_1^* > r_2^* + r_3^*$ is satisfied, and the optimum weights in this case should satisfy $w_1 > w_2 + w_3$, i.e. $w_1 > 0.5$. Therefore, the expert rule governed by individual 1 is the optimal decision-making method. The resulting mean performance is indeed just his/her ability p_1. Similarly, the second and third cases mean that individuals 2 and 3 are so excellent that the expert rule governed by individuals 2 or 3 is optimal, respectively. In the fourth case, none of them is excellent, or none of the log-odds ratios exceeds the sum of the other two's. Any weight should be less than 0.5 in this case. Whenever the set of weights holds such inequalities, the outcome of the weighted majority vote is equal to that of the unweighted majority vote, because the sum of the two weights is always greater than the other one. In this case, the mean performance becomes M, which is the accuracy of the result of the unweighted majority vote among these three individuals; M is the sum of the probabilities that two or three, i.e., the majority of the primary choices, are correct.

A summary of the optimum weights and mean performance is also shown in Fig. 1 in the main text or Fig. S.1, where 2-simplex $\{\mathbf{w}; w_1 + w_2 + w_3 = 1\}$ is divided into four domains according to the optimum weight conditions.

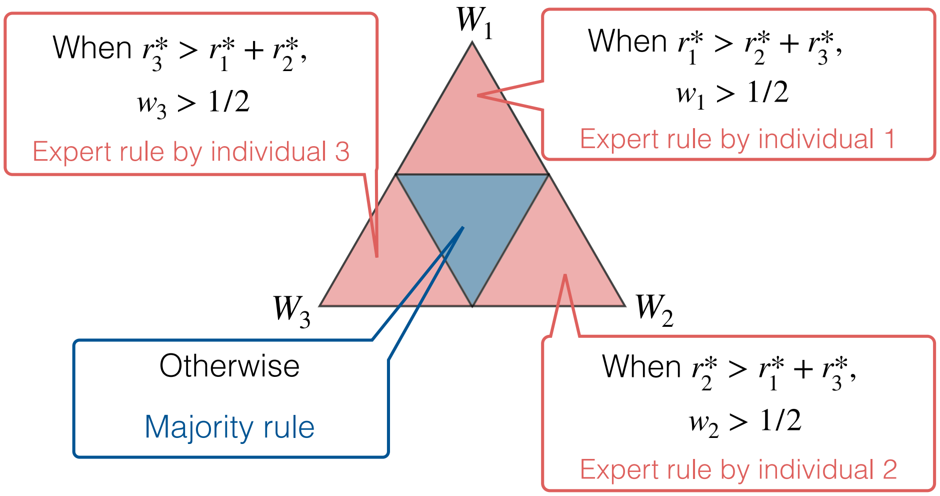

Figure S.1: Summary of the region of optimum weights w_n of individual n ($n = 1, 2, 3$) in simultaneous decision-making involving three individuals. The largest triangle exhibits the 2-simplex $\{\mathbf{w} = (w_1, w_2, w_3)\,; w_1 + w_2 + w_3 = 1\}$, where W_1, W_2, and W_3 denote $(1, 0, 0)$, $(0, 1, 0)$, and $(0, 0, 1)$, respectively. Each smaller triangle exhibits the region of optimum weights when each inequality for r_1^*, r_2^*, and r_3^* holds. For example, when the ability of individual 1 is so high that $r_1^* > r_2^* + r_3^*$ is satisfied, the optimum weights should satisfy $w_1 > 0.5$, i.e. $w_2 + w_3 < 0.5$,, which corresponds to the upper red triangle, and these weights correspond to the expert rule governed by individual 1.

S.2 Sequential decision-making

Hereafter, we consider sequential decision-making, where N persons sequentially answer a binary choice problem, as explained in Section 2.1 in the main text. The primary choice of the n-th respondent is denoted by X_n. He/she can observe the answers $(Y_1, ..., Y_{n-1})$ given by his/her antecedents and holds an optimally weighted majority vote among $Y_1, ..., Y_{n-1}$ and X_n to maximise his/her conditional performance, given the answers of these antecedents and his/her primary choice, where Y_m is the answer of the m-th respondent. The n-th respondent finally answers Y_n, which is the outcome of the weighted majority vote. The ability of the n-th respondent is denoted by p_n.

S.2.1 Casting vote theorem and optimal behaviour

Here, we show the derivation of the optimal behaviour for each respondent to maximise his/her conditional performance, given his/her antecedents' answers and primary choice (Section 3.2 in the main text). We also provide a proof of the casting vote theorem in Section 3.1 in the main text. As described in the main text, we call the primary choice of a respondent *casting vote* when his/her answer is determined only by his/her primary choice, given an opinion distribution of the antecedents' answers. In addition, we call a respondent a casting voter when her primary choice is a casting vote.

First respondent's decision-making

The first respondent is always a casting voter because he/she decides independently by definition. Hereafter, let us denote the alternative answered by the first respondent as s and the opposite answer as t. We consider three subsets S_n, T_n and R_n of the set $\{1, 2, ..., n\}$, where S_n is a set of indices of respondents (from the first to the n-th respondents) whose primary choices were casting votes and who answered s; T_n is a set of indices whose primary choices were casting votes and who answered t; and R_n is a set of indices whose primary choices were not casting votes. Thus, $\{1\}$ is defined as S_1. Both T_1 and R_1 are empty.

The n-th respondent

Subsequently, we consider decision-making by the n-th respondent ($n > 1$) in each of the following two cases: Case 1) the indices of her antecedents are separated into S_{n-1} and T_{n-1}, and $R_{n-1} = \phi$; Case 2) the antecedents' indices are separated into S_{n-1}, T_{n-1}, and $R_{n-1}(\neq \phi)$.

Case 1) Antecedents' indices are separated into S_{n-1} and T_{n-1}

First, we assume that the indices of the n-th respondents' antecedents are separated only by S_{n-1} and T_{n-1} ($S_{n-1} \sqcup T_{n-1} = \{1, 2, ..., n-1\}$, $R_{n-1} = \phi$). The second respondent always faces this situation. As all the antecedents of the n-th respondent are casting voters, $Y_1, Y_2, ..., Y_{n-2}$ and Y_{n-1} are independent of each other. In addition, the primary

choice X_n of the n-th respondent is independent of the answers of his/her antecedents. Therefore, for the n-th respondent, we can calculate the probability of having the answers and primary choice $(Y_1, ..., Y_{n-1}, X_n)$ when one alternative is correct, which is also regarded as the likelihood that the alternative is correct, given this opinion distribution, in the same manner as that for simultaneous decision-making in Section S.1.

When the primary choice of the n-th respondent is s, the likelihood of s being correct, given $(Y_1, ..., Y_{n-1}, X_n = s)$ is $P(S_{n-1})Q(T_{n-1})p_n$, and the likelihood that t is correct is $Q(S_{n-1})P(T_{n-1})(1 - p_n)$. Based on the same discussion as that for simultaneous decision-making (Section S.1), the n-th respondent should answer s with probability 1 if $P(S_{n-1})Q(T_{n-1})p_n > Q(S_{n-1})P(T_{n-1})(1 - p_n)$, i.e., $r_n^* + \sum_{m \in S_{n-1}} r_m^* > \sum_{m \in T_{n-1}} r_m^*$ to maximise the conditional performance given these answers and the primary choice. By contrast, he/she should answer t with probability 1 if $r_n^* + \sum_{m \in S_{n-1}} r_m^* < \sum_{m \in T_{n-1}} r_m^*$. The conditional performance $\pi_n(S_{n-1}, T_{n-1}, X_n = s)$ of the n-th respondent is

$$\pi_n(S_{n-1}, T_{n-1}, X_n = s) = \max \left[\frac{P(S_{n-1})Q(T_{n-1})p_n}{Z_{S_{n-1},T_{n-1},X_n=s}}, \ \frac{Q(S_{n-1})P(T_{n-1})(1 - p_n)}{Z_{S_{n-1},T_{n-1},X_n=s}} \right], \quad \text{(S.10)}$$

where $Z_{S_{n-1},T_{n-1},X_n=s} = P(S_{n-1})Q(T_{n-1})p_n + Q(S_{n-1})P(T_{n-1})(1 - p_n)$, using Eq. (S.6).

Similarly, when $X_n = t$, the likelihood of s being correct given $(Y_1, ..., Y_{n-1}, X_n = t)$ is $P(S_{n-1})Q(T_{n-1})(1 - p_n)$ and the likelihood that t is correct is $Q(S_{n-1})Q(T_{n-1})p_n$. The n-th respondent should answer s with probability 1 if $\sum_{m \in S_{n-1}} r_m^* > r_n^* + \sum_{m \in T_{n-1}} r_m^*$ and answer t with probability 1 if $\sum_{m \in S_{n-1}} r_m^* < r_n^* + \sum_{m \in T_{n-1}} r_m^*$ to maximise the conditional

performance given the answers and primary choice. The resulting conditional performance $\pi_n(S_{n-1}, T_{n-1}, X_n = t)$ is

$$\pi_n(S_{n-1}, T_{n-1}, X_n = t) = \max\left[\frac{P(S_{n-1})Q(T_{n-1})(1-p_n)}{Z_{S_{n-1},T_{n-1},X_n=t}},\ \frac{Q(S_{n-1})Q(T_{n-1})p_n}{Z_{S_{n-1},T_{n-1},X_n=t}}\right], \quad \text{(S.11)}$$

where $Z_{S_{n-1},T_{n-1},X_n=t} = P(S_{n-1})Q(T_{n-1})(1-p_n) + Q(S_{n-1})Q(T_{n-1})p_n$. To avoid complications in the treatment of ties, we do not consider $\{p_n\}$ such that $r_n^* + \sum_{m \in S_{n-1}} r_m^* = \sum_{m \in T_{n-1}} r_m^*$ or $\sum_{m \in S_{n-1}} r_m^* = r_n^* + \sum_{m \in T_{n-1}} r_m^*$ are satisfied.

Here, we summarise the optimal behaviour of the n-th respondent given the opinion distribution (S_{n-1}, T_{n-1}) of the antecedents based on the relationship between the abilities of the antecedents and the respondent; more precisely, based on the relationship between $r_n^* + \sum_{m \in S_{n-1}} r_m^*$ and $\sum_{m \in T_{n-1}} r_m^*$ or $\sum_{m \in S_{n-1}} r_m^*$ and $r_n^* + \sum_{m \in T_{n-1}} r_m^*$, as in the following four cases:

1. Case of

$$r_n^* + \sum_{m \in S_{n-1}} r_m^* > \sum_{m \in T_{n-1}} r_m^* \text{ and } \sum_{m \in S_{n-1}} r_m^* > r_n^* + \sum_{m \in T_{n-1}} r_m^*. \quad \text{(S.12)}$$

In this case, the n-th respondent answers s with probability 1 when $X_n = s$, and answers t with probability 1 when $X_n = t$, as his/her optimal behaviour (see rules A) and B) in Section S.1.1). Therefore, the respondent should answer s with probability 1 irrespective of his/her primary choice. Thus, his/her primary choice is not a casting

vote, and n is included in R_n ($R_n = R_{n-1} \cup \{n\}$). The probability of finally observing the answers by the first to the n-th respondents can be written as

- $P(S_{n-1})Q(T_{n-1}) \times 1 = P(S_{n-1})Q(T_{n-1})$ when s is correct.
- $Q(S_{n-1})P(T_{n-1})$ when t is correct.

2. Case of

$$r_n^* + \sum_{m \in S_{n-1}} r_m^* < \sum_{m \in T_{n-1}} r_m^* \text{ and } \sum_{m \in S_{n-1}} r_m^* < r_n^* + \sum_{m \in T_{n-1}} r_m^*. \tag{S.13}$$

By a similar discussion of the first case, the n-th respondent answers t with probability 1 irrespective of his/her primary choice, as his/her optimal behaviour (see rules A) and B) in Section S.1.1). Therefore, his/her primary choice is not the casting vote ($R_n = R_{n-1} \cup \{n\}$). The probability of finally observing a set of answers by the first to the n-th respondents can be represented as

- $P(S_{n-1})Q(T_{n-1})$ when s was correct.
- $Q(S_{n-1})P(T_{n-1})$ when t was correct.

3. Case of

$$r_n^* + \sum_{m \in S_{n-1}} r_m^* > \sum_{m \in T_{n-1}} r_m^* \text{ and } \sum_{m \in S_{n-1}} r_m^* < r_n^* + \sum_{m \in T_{n-1}} r_m^*. \tag{S.14}$$

The n-th respondent answers s when her primary choice is s and answers t when her

primary choice is t, as her optimal behaviour (see rules A) and B) in Section S.1.1). This means that her answer is her primary choice. Therefore, her primary choice is a casting vote. Thus, n is included in S_n or T_n according to her primary choice. Given the opinion distribution (S_{n-1}, T_{n-1}) of answers by the antecedents, the conditional probability that the n-th respondent answers s is p_n and that she answers t is $1-p_n$ when s is correct. When t is correct, the conditional probability of answering s is $1-p_n$ and that of answering t is p_n. Therefore, the probability of finally observing a set of answers by the first to the n-th respondents can be written as

- $P(S_{n-1})Q(T_{n-1})p_n$ when $X_n = s$ and s was correct.
- $P(S_{n-1})Q(T_{n-1})(1-p_n)$ when $X_n = t$ and s was correct.
- $Q(S_{n-1})P(T_{n-1})(1-p_n)$ when $X_n = s$ and t was correct.
- $Q(S_{n-1})P(T_{n-1})p_n$ when $X_n = t$ and t was correct.

4. Case of

$$r_n^* + \sum_{m \in S_{n-1}} r_m^* < \sum_{m \in T_{n-1}} r_m^* \text{ and } \sum_{m \in S_{n-1}} r_m^* > r_n^* + \sum_{m \in T_{n-1}} r_m^* \tag{S.15}$$

cannot occur as $p_n > 0.5$ and $r_n^* > 0$.

Here, the conditions of $r_n^* + \sum_{m \in S_{n-1}} r_m^* > \sum_{m \in T_{n-1}} r_m^*$ and $\sum_{m \in S_{n-1}} r_m^* > r_n^* + \sum_{m \in T_{n-1}} r_m^*$ in the first case can be reduced to only $\sum_{m \in S_{n-1}} r_m^* > r_n^* + \sum_{m \in T_{n-1}} r_m^*$ because the former inequality is always satisfied when the latter inequality is satisfied. Similarly, in the second case, the conditions of $r_n^* + \sum_{m \in S_{n-1}} r_m^* < \sum_{m \in T_{n-1}} r_m^*$ and $\sum_{m \in S_{n-1}} r_m^* <$

$r_n^* + \sum_{m \in T_{n-1}} r_m^*$ can be reduced to only $r_n^* + \sum_{m \in S_{n-1}} r_m^* < \sum_{m \in T_{n-1}} r_m^*$. Under the third case, $r_n^* + \sum_{m \in S_{n-1}} r_m^* > \sum_{m \in T_{n-1}} r_m^*$ and $\sum_{m \in S_{n-1}} r_m^* < r_n^* + \sum_{m \in T_{n-1}} r_m^*$ means $r_n^* > \left| \sum_{i \in S_{n-1}} r_m^* - \sum_{m \in T_{n-1}} r_m^* \right|$,

This summary of optimal behaviour according to the relationship between the respondents' abilities is incorporated into Table 1 in the main text (Section 3.2).

In summary, the n-th respondent should either answer regardless of his/her primary choice or always answer his/her primary choice, and n is assigned to either R_n, S_n, or T_n given that the respondent's antecedents are separated into S_{n-1} and T_{n-1}. The probability of finally observing the answers of the first to the n-th respondents can also be summarised as follows. When s is correct, this probability is expressed as

$$P(S_{n-1})Q(T_{n-1})P_n, \tag{S.16}$$

where

$$P_n = \begin{cases} 1, \text{ if } n \in R_n, \\ p_n, \text{ if } n \in S_n, \\ 1 - p_n, \text{ if } n \in T_n. \end{cases} \tag{S.17}$$

When t is correct, this probability is expressed as

$$Q(S_{n-1})P(T_{n-1})P'_n, \tag{S.18}$$

where

$$P_n' = \begin{cases} 1, \text{ if } n \in R_n, \\ 1 - p_n, \text{ if } n \in S_n, \\ p_n, \text{ if } n \in T_n. \end{cases} \tag{S.19}$$

Case 2) Antecedents are separated into S_{n-1}, T_{n-1} and R_{n-1}

Thus far, we have shown that by the optimal behaviour of the m-th respondent, m can be assigned to either S_m, T_m, or R_m provided that the antecedents' indices are separated into either S_{m-1} or T_{m-1} ($R_{m-1} = \phi$). We now consider decision-making by the n-th respondent when the antecedents' indices are separated into S_{n-1}, T_{n-1}, and $R_{n-1}(\neq \phi)$.

From the discussion in *Case 1*, the probability of observing the answers by the antecedents given the separation of $(S_{n-1}, T_{n-1}, R_{n-1})$ can be represented as $P(S_{n-1})Q(T_{n-1}) \prod_{m \in R_{n-1}} 1 = P(S_{n-1})Q(T_{n-1})$ when s is correct and $Q(S_{n-1})P(T_{n-1}) \prod_{m \in R_{n-1}} 1 = Q(S_{n-1})P(T_{n-1})$ when t is correct. Therefore, given the opinion distribution $(S_{n-1}, T_{n-1}, R_{n-1})$, the probability of observing the answers by the antecedents can be written only by the abilities of antecedents in S_{n-1} and T_{n-1} as well as that in *Case 1*. The optimal behaviour for the n-th respondent in *Case 2* is the same as that shown in *Case 1* if we substitute $\pi_n(S_{n-1}, T_{n-1}, X_n = s)$, $\pi_n(S_{n-1}, T_{n-1}, X_n = t)$, $Z_{S_{n-1},T_{n-1},X_n=s}$ and $Z_{S_{n-1},T_{n-1},X_n=t}$ to $\pi_n(S_{n-1}, T_{n-1}, R_{n-1}, X_n = s)$, $\pi_n(S_{n-1}, T_{n-1}, R_{n-1}, X_n = t)$, $Z_{S_{n-1},T_{n-1},R_{n-1},X_n=s}$ and $Z_{S_{n-1},T_{n-1},R_{n-1},X_n=t}$, respectively. The probability of finally observing the answers of the first to the n-th respondents can be represented as follows in

the same manner as in *Case 1*:

- $P(S_{n-1})Q(T_{n-1})p_n$ when $X_n = s$ and s was correct.

- $P(S_{n-1})Q(T_{n-1})(1-p_n)$ when $X_n = t$ and s was correct.

- $Q(S_{n-1})P(T_{n-1})(1-p_n)$ when $X_n = s$ and t was correct.

- $Q(S_{n-1})P(T_{n-1})p_n$ when $X_n = t$ and t was correct.

Finally, for the n-th respondent, the mean performance $\mathrm{E}[\pi_n(S_{n-1}, T_{n-1}, R_{n-1}, X_n)]$ over the possible opinion distribution $(S_{n-1}, T_{n-1}, R_{n-1})$ of the antecedents and primary choice X_n can be written as:

$$
\begin{aligned}
&\mathrm{E}[\pi_n(S_{n-1}, T_{n-1}, R_{n-1}, X_n)] \\
&= \sum_{(S_{n-1},T_{n-1},R_{n-1})} \Big[Z_{S_{n-1},T_{n-1},R_{n-1},X_n=s} \pi_n(S_{n-1}, T_{n-1}, R_{n-1}, X_n = s) \\
&\qquad\qquad + Z_{S_{n-1},T_{n-1},R_{n-1},X_n=t} \pi_n(S_{n-1}, T_{n-1}, R_{n-1}, X_n = t) \Big] \\
&= \sum_{(S_{n-1},T_{n-1},R_{n-1})} \Big[\max\left[P(S_{n-1})Q(T_{n-1})p_n, Q(S_{n-1})P(T_{n-1})(1-p_n)\right] \\
&\qquad\qquad + \max\left[P(S_{n-1})Q(T_{n-1})(1-p_n), Q(S_{n-1})P(T_{n-1})p_n\right] \Big]. \qquad \text{(S.20)}
\end{aligned}
$$

S.2.2 Sequential decision-making of three individuals

Here, we show in detail the optimal behaviour of the second and third respondents in sequential decision-making, as shown in Section 3.3 in the main text.

Optimal behaviour of the second respondent

First, we considered decision-making by the second respondent. As his/her primary choice X_2 is independent of the answer Y_1 by the first respondent, his/her optimal behaviour can be determined by applying the calculation of that in the case of simultaneous decision-making involving two individuals (Section S.1.2). When $p_1 > p_2$, the expert rule governed by the answer of the first respondent is optimal in the decision-making of the second respondent. Therefore, Y_2 should always be the same as Y_1 and $2 \in R_2$. When $p_1 < p_2$, the respondent should always give his/her primary choice X_2 as the answer, i.e., he/she should be a casting voter, and 2 is included in either S_2 or T_2 according to his/her primary choice.

Optimal behaviour of the third respondent

Subsequently, we considered decision-making by the third respondent. When $p_1 < p_2$, the second respondent is a casting voter ($2 \in S_2$ or $2 \in T_2$). Therefore, Y_1, Y_2 and X_3 are independent of one another. Thus, when $p_1 < p_2$, the optimal behaviour of the third respondent can be determined in the same manner as that for the simultaneous decision-making involving three individuals (Section S.1.3). Hereafter, we determine the optimal behaviour of the third respondent when $p_1 > p_2$ and $2 \in R$.

When the primary choice X_3 of the third respondent is s, which is the same as the first choice, the third respondent should always answer s because $r_1^* + r_3^* > 0$. We do not have to consider r_2^* because $2 \in R_2$. Based on this optimal behaviour, the conditional

performance $\pi_3(S_2, T_2, R_2, X_3 = s)$ becomes:

$$\pi_3(S_2, T_2, R_2, X_3 = s) = \frac{p_1 p_3}{p_1 p_3 + (1 - p_1)(1 - p_3)}, \tag{S.21}$$

using Eq. (S.10). When $X_3 = t$, the respondent should answer s if $r_1^* > r_3^*$, i.e., $p_1 > p_3$, and answer t if $r_1^* < r_3^*$, i.e., $p_1 < p_3$. Then, the conditional performance becomes

$$\pi_3(S_2, T_2, R_2, X_3 = t) = \max\left[\frac{p_1(1 - p_3)}{p_1(1 - p_3) + (1 - p_1)p_3}, \frac{(1 - p_1)p_3}{p_1(1 - p_3) + (1 - p_1)p_3}\right], \tag{S.22}$$

by Eq. (S.11).

Therefore, when $p_1 > p_2$, the mean performance $\mathrm{E}[\pi_3(S_2, T_2, R_2, X_3)]$ of the third respondent can be written as

$$\begin{aligned}
\mathrm{E}[\pi_3(S_2, T_2, R_2, X_3)] &= \mathrm{E}\left[\pi_3\left(S_2 = \{1\}, T_2 = \phi, R_2 = \{2\}, X_3\right)\right] \\
&= \sum_{X_3} Z_{S_2=\{1\}, T_2=\phi, R_2=\{2\}, X_3} \pi_3(S_2 = \{1\}, T_2 = \phi, R_2 = \{2\}, X_3) \\
&= p_1 p_3 + \max\left[p_1(1 - p_3), (1 - p_1)p_3\right], \qquad \text{(S.23)}
\end{aligned}$$

which is p_1 when $p_1 > p_3$ and p_3 when $p_1 < p_3$.

S.2.3 Sequential decision-making of individuals with the same ability

S.2.3.1 Arbitrary number of persons with the same ability

In this section, we assume the sequential decision-making of individuals with the same ability p as shown in Section 3.3 of the main text.

We consider decision-making by the n-th respondent. Let S_{n-1} and T_{n-1} be sets of indices of the antecedents that were casting voters and answered s and t, respectively, as explained in the previous section. In addition, let R_{n-1} denote the set of antecedents that are not casting voters. $|X|$ denotes the number of elements in the set X.

We can use the algorithm shown in Table 1 in the main text to determine the optimal behaviour of each individual even when all individuals have the same ability for the following reason. In simultaneous decision-making (Section S.1.1), the probability of having an opinion distribution (S, T) of individuals whose abilities are p can be written as $Cp^{|S|}(1-p)^{|T|}$ if s is correct, and $C'(1-p)^{|S|}p^{|T|}$ if t is correct. Here, coefficients C and C' are identical: $C =_{|S|+|T|} C_{|S|} =_{|S|+|T|} C_{|T|} = C'$. Therefore, the likelihood that s is correct is greater than that of t when and only when $|S|r^* > |T|r^*(\Leftrightarrow |S| > |T|)$, where $r^* = \log\left[p/(1-p)\right](> 0)$. Similarly, the likelihood of t being correct is greater than the others when and only when $|S| < |T|$. We can incorporate this calculation into the discussion of sequential decision-making, as shown in Section S.2.1. However, as noted in the main text, we additionally assume that the n-th respondent chooses his/her primary

choice if either choice is equally likely to be true.

The optimum answer Y_n for the n-th respondent can be derived, as shown in Table 1 in the main text. When $X_n = s$,

$$Y_n|_{X_n=s} = \begin{cases} s, & \text{if } |S_{n-1}| + 1 \geq |T_{n-1}|, \\ t, & \text{if } |S_{n-1}| + 1 < |T_{n-1}|, \end{cases} \tag{S.24}$$

and, when $X_n = t$,

$$Y_n|_{X_n=t} = \begin{cases} s, & \text{if } |S_{n-1}| \geq |T_{n-1}| + 1, \\ t, & \text{if } |S_{n-1}| < |T_{n-1}| + 1. \end{cases} \tag{S.25}$$

The difference $d_n = |S_{n-1}| - |T_{n-1}|$ in votes for s and t in the preceding casting votes for the n-th respondent can be regarded as a random walk on integer steps from -2 to 2. The difference d_2 is 1 by definition, as the first respondent, who is unconditionally a casting voter, answered s. If the second respondent's primary choice is s, he/she should choose s according to (S.24), while if his/her primary choice is t, he/she chooses t because s and t are equally likely based on the opinion distribution. Hence, the second respondent is a casting voter, and d_3 will be either 2 or 0 depending on the second respondent's primary choice. It will be $d_3 = 2$ if the second respondent's primary choice is s. If this is the case, the third and all subsequent respondents are all non-casting voters answering s. In addition, the difference d stays in 2 until the end of the sequential decision ($d_n = 2$, $n = 3, 4, ..., N$) because n is included in R_n when the n-th respondent observes $|d_n| = 2$ in

the answers given by his/her antecedents. Conversely, R_{n-1} should be empty when $|d_n| < 2$ due to the following reason: if R_{n-1} included $m(< n-1)$, this means that $|d_m| = 2$, which leads to a contradiction where $|d_n| = 2$. The state 2 is therefore an absorbing state of the random walk. For the same reason, $d_n = -2$ is the absorbing state. By contrast, if $d_3 = 0$, is chosen, a similar inspection of (S.24)–(S.25) reveals that the third respondent is a casting voter, and d_4 becomes either 1 or -1 depending on whether his/her primary choice is s or t. This consideration is sufficient to describe the behaviour of the process. In summary, the n-th respondent becomes a casting voter when $|d_n| = 1$ or $|d_n| = 0$, whereas he/she becomes a non-casting voter when $|d_n| = 2$.

Considering the optimal behaviour by each respondent discussed so far, we can describe how correct and wrong answers increase in sequential decision-making. Figures S.2 and S.3 show the number of correct and incorrect answers in the sequential decision-making involving five and six individuals, respectively. For example, the thick lines in Fig. S.3 stand for the following situation: the first respondent answered the correct alternative; the second respondent was a casting voter as $d_2 = 1$ among the answers given by his/her antecedents and he/she gave a wrong answer; the third respondent was a casting voter as $d_3 = 0$ and he/she answered correctly; the fourth respondent was a casting voter as $d_4 = 1$ among the answers given by his/her antecedents and he/she answered correctly; the fifth respondent answered correctly regardless of his/her primary choice as $d_5 = 2$; the sixth respondent then observed $S_5 = \{1, 3, 4\}$, $T_5 = \{2\}$, and $R_5 = \{5\}$ and answered correctly regardless of his/her primary choice as $d_6 = 2$. In this case, respondents later than the

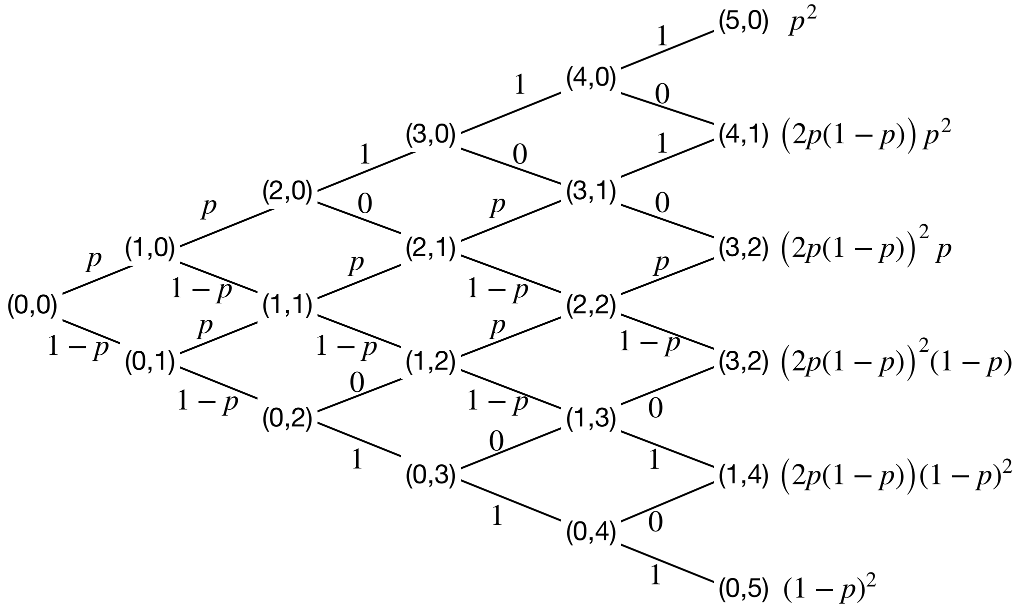

Figure S.2: Number of correct and wrong answers in the sequential decision-making involving five individuals. The pair (x, y) at the m-th column from the left-hand side exhibits the number of correct (x) and wrong (y) answers in the answers given by the antecedents of the m-th respondent. Transition probability between two states of answers is shown on the line between two brackets. The probability that the sixth respondent will observe each state of answers is shown on the right-hand side.

fourth respondent do not answer incorrectly.

Let us first assume that n is odd, i.e. $n = 2k-1$ $(k = 1, 2, ...)$. We derive the conditional and mean performance of the n-th respondent in this case. The conditional performance of the n-th respondent given $(S_{n-1}, T_{n-1}, R_{n-1})$ in the answers of his/her antecedents and primary choice, $\pi_n(S_{n-1}, T_{n-1}, R_{n-1}, X_n)$, is described as follows. When s is correct,

- if $d_n = |S_{n-1}| - |T_{n-1}| = 2$ $(|T_{n-1}| = 0, 1, ..., k-2)$, the probability of observing the answers given by the antecedents of the n-th respondent is $[2p(1-p)]^{|T_{n-1}|}\, p^2$. The respondent answers s regardless of his/her primary choice as his/her optimal

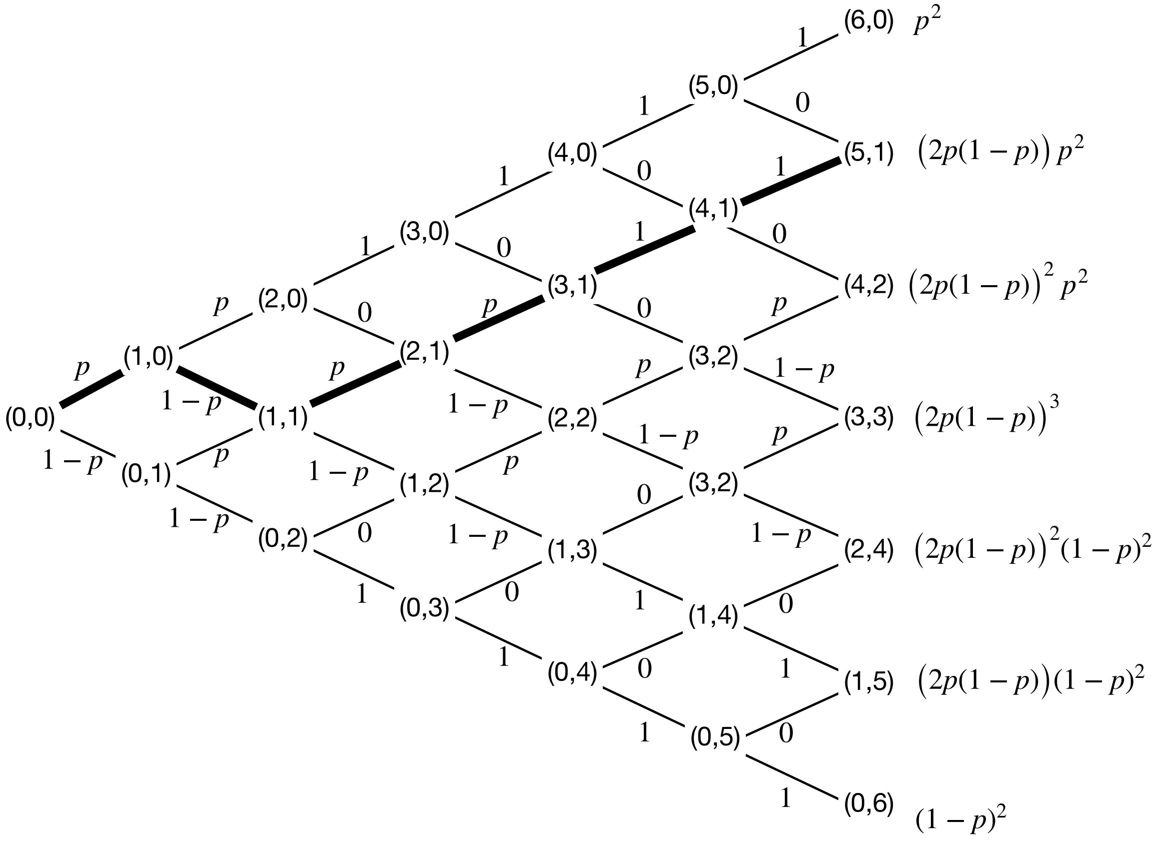

Figure S.3: Number of correct and wrong answers in the sequential decision-making involving six individuals. The meaning of the parentheses is the same as that in Fig. S.2.

behaviour and conditional performance $\pi_n(S_{n-1}, T_{n-1}, R_{n-1}, X_n)$ is 1.

- If $d_n = 0$, i.e., $|S_{n-1}| = |T_{n-1}| = k-1$, the probability of observing the answers given by the antecedents of the n-th respondent is $[2p(1-p)]^{k-1}$. The respondent becomes a casting voter as his/her optimal behaviour, and $\pi_n(S_{n-1}, T_{n-1}, R_{n-1}, X_n) = p$.

- If $-d_n = |T_{n-1}| - |S_{n-1}| = 2$ ($|S_{n-1}| = 0, 1, ..., k-2$), the probability of observing the answers given by antecedents of the n-th respondent is $[2p(1-p)]^{|T_{n-1}|}\,(1-p)^2$. The respondent answers t as his/her optimal behaviour, and $\pi_n(S_{n-1}, T_{n-1}, R_{n-1}, X_n) =$

0.

Here, d_n cannot be 1 when n is odd, for the following reason. Now, $|S_{n-1}|+|T_{n-1}|+|R_{n-1}|$ is even as $n = |S_{n-1}|+|T_{n-1}|+|R_{n-1}|+1$ is odd. If d_n was 1, R_{n-1} should be empty because $d_n < 2$ as discussed previously. Therefore, $|S_{n-1}| + |T_{n-1}|$ equals $|S_{n-1}| + |T_{n-1}| + |R_{n-1}|$ and is even. Thus, $d_n = |S_{n-1}| - |T_{n-1}|$ should be even, which contradicts $d_n = 1$. When t is correct,

- if $d_n = |S_{n-1}| - |T_{n-1}| = 2$ ($|T_{n-1}| = 0, 1, ..., k-2$), the probability of observing the answers given by the antecedents of the n-th respondent is $[2p(1-p)]^{|T_{n-1}|}(1-p)^2$. The respondent answers s regardless of his/her primary choice as his/her optimal behaviour, and $\pi_n(S_{n-1}, T_{n-1}, R_{n-1}, X_n) = 0$.

- If $d_n = 0$, i.e., $|S_{n-1}| = |T_{n-1}| = k-1$, the probability of observing the answers given by the antecedents of the n-th respondent is $[2p(1-p)]^{k-1}$. His/her optimal behaviour is giving his/her primary choice as the answer, and $\pi_n(S_{n-1}, T_{n-1}, R_{n-1}, X_n) = p$.

- If $-d_n = |T_{n-1}| - |S_{n-1}| = 2$ ($|S_{n-1}| = 0, 1, ..., k-2$), the probability of observing the answers given by the antecedents of the n-th respondent is $[2p(1-p)]^{|T_{n-1}|}p^2$. The respondent answers t regardless of his/her primary choice as his/her optimal behaviour, and $\pi_n(S_{n-1}, T_{n-1}, R_{n-1}, X_n) = 1$.

We further investigate the mean performance of the n-th respondent as n is odd. The probability that s is correct is p and that t is correct is $1-p$ because the ability of

the first respondent is p. Therefore, the mean of the conditional performance $\mathrm{E}[\pi_n] := \mathrm{E}[\pi_n(S_{n-1}, T_{n-1}, R_{n-1}, X_n)]$ over the possible $(S_{n-1}, T_{n-1}, R_{n-1}, X_n)$, simply called the mean performance, is

$$\begin{aligned}
\mathrm{E}[\pi_n] = p & \left[\sum_{|T_{n-1}|=0}^{k-2} [2p(1-p)]^{|T_{n-1}|} p^2 + [2p(1-p)]^{k-1} p \right] \\
& + (1-p) \left[\sum_{|S_{n-1}|=0}^{k-2} [2p(1-p)]^{|S_{n-1}|} p^2 + [2p(1-p)]^{k-1} p \right] \\
& = pQ^n_{2|s} + pQ^n_{\{-1,0,1\}} + (1-p)Q_{-2|t},
\end{aligned} \tag{S.26}$$

where $Q^n_{2|s} = \sum_{|T_{n-1}|=0}^{k-2} [2p(1-p)]^{|T_{n-1}|} p^2$, $Q^n_{\{-1,0,1\}} = \sum_{|T_{n-1}|=0}^{k-2} [2p(1-p)]^{k-1} p$, and $Q^n_{2|t} = \sum_{|S_{n-1}|=0}^{k-2} [2p(1-p)]^{|S_{n-1}|} p^2$ for $k \geq 2$, and $Q^n_{2|s} = Q^n_{2|t} = 0$ for $k = 1$. Note that both $Q^n_{2|s}$ and $Q^n_{-2|t}$ can be written as $\sum_{m=0}^{k-2} [2p(1-p)]^m p^2$ by substituting the indices $|S_{n-1}|$ or $|T_{n-1}|$ into m.

Equation (S.26) is explained as follows. With the probability $p \times Q^n_{2|s}$, s is correct, and the difference in the votes for s and t by casting voters is 2 ($d_n = 2$). The n-th respondent then unconditionally answers s, which is correct in this case. This yields the first term in Eq. (S.26). The last term of Eq. (S.26) is given similarly: with probability $(1-p)Q^n_{-2|t}$, t is correct, and the difference in the votes for s and t of the casting voters is -2 ($d_n = -2$). The n-th respondent then unconditionally answers t, which is correct. Finally, if d_n is either -1, 0, or 1, the probability of which is $Q^n_{\{-1,0,1\}}$, then the n-th respondent answers his/her primary choice as he/she is a casting voter. The accuracy of his/her answer is

therefore p, which gives the second term of (S.26).

Subsequently, we assume that n is even, i.e. $n = 2k$ $(k = 1, 2, ...)$. In this case, $|S_{n-1}| + |T_{n-1}|$ is odd, and d_n cannot be 0 by a similar discussion for the case where n is odd. Here, we must consider the conditional performance of the n-th respondent when $|d_n| = 1$, which is not the case when n is even. When s was correct,

- if $|d_n| = 2$, the conditional performance of the n-th respondent $\pi_n(S_{n-1}, T_{n-1}, R_{n-1}, X_n)$ given opinion distribution $(S_{n-1}, T_{n-1}, R_{n-1})$ among his/her antecedents, and his/her primary choice is the same as that in the case where n is odd.

- If $d_n = |S_{n-1}| - |T_{n-1}| = 1$ $(|T_{n-1}| = k - 1)$, the probability of observing the answers given by the antecedents of the n-th respondent is $[2p(1-p)]^{k-1} p$. His/her optimal behaviour is giving his/her primary choice as her answer, and $\pi_n(S_{n-1}, T_{n-1}, R_{n-1}, X_n) = p$.

- If $-d_n = |T_{n-1}| - |S_{n-1}| = 1$ $(|S_{n-1}| = k - 1)$, the probability of observing the answers given by the antecedents of the n-th respondent is $[2p(1-p)]^{k-1} (1-p)$. His/her optimal behaviour is giving his/her primary choice as the answer, and $\pi_n(S_{n-1}, T_{n-1}, R_{n-1}, X_n) = p$.

When t was correct,

- if $|d_n| = 2$, the conditional performance is the same as that in the case where n is odd.

- If $d_n = |S_{n-1}| - |T_{n-1}| = 1$ ($|T_{n-1}| = k-1$), the probability of observing the answers given by the antecedents of the n-th respondent is $[2p(1-p)]^{k-1}(1-p)$. His/her optimal behaviour is giving his/her primary choice as the answer, and $\pi_n(S_{n-1}, T_{n-1}, R_{n-1}, X_n) = p$.

- If $-d_n = |T_{n-1}| - |S_{n-1}| = 1$ ($|S_{n-1}| = k-1$), the probability of observing the answers given by the antecedents of the n-th respondent is $[2p(1-p)]^{k-1}p$. His/her optimal behaviour is giving his/her primary choice as the answer, and $\pi_n(S_{n-1}, T_{n-1}, R_{n-1}, X_n) = p$.

The mean performance $\mathrm{E}[\pi_n]$ of the n-th respondent as n is even is

$$\begin{aligned}
\mathrm{E}[\pi_n] = p &\left[\sum_{|T_{n-1}|=0}^{k-2} [2p(1-p)]^{|T_{n-1}|} p^2 + \left[[2p(1-p)]^{k-1} p + [2p(1-p)]^{k-1}(1-p) \right] p \right] \\
&+ (1-p) \left[\sum_{|S_{n-1}|=0}^{k-2} [2p(1-p)]^{|S_{n-1}|} p^2 + \left[[2p(1-p)]^{k-1}(1-p) + [2p(1-p)]^{k-1} p \right] p \right] \\
&= pQ^n_{2|S} + pQ^n_{\{-1,0,1\}} + (1-p)Q_{-2|t}, \qquad \text{(S.27)}
\end{aligned}$$

which is the same as in Eq. (S.26).

In summary, for both $n = 2m-1$ and $n = 2m$ ($m \geq 1$), the mean performance of the

n-th respondent is

$$\begin{aligned}
\mathrm{E}[\pi_n] &= pQ^n_{2|s} + pQ^n_{\{-1,0,1\}} + (1-p)Q_{-2|t} \\
&= \sum_{m=0}^{k-2}[2p(1-p)]^m p^2 + [2p(1-p)]^{k-1}p \\
&= \frac{1-(2p(1-p))^{k-1}}{1-(2p(1-p))}p^2 + (2p(1-p))^{k-1}p \\
&= \frac{p^2 + p(1-p)(1-2p)\,(2p(1-p))^{k-1}}{p^2+(1-p)^2} \\
&\xrightarrow[k\to\infty]{} \frac{p^2}{p^2+(1-p)^2} \;\; (:= \pi_{\max}(p)). \qquad \text{(S.28)}
\end{aligned}$$

The first few values of $\mathrm{E}[\pi_n]$ are

$$\mathrm{E}[\pi_1] = \mathrm{E}[\pi_2] = p, \qquad \text{(S.29)}$$

$$\mathrm{E}[\pi_3] = \mathrm{E}[\pi_4] = p^2(3-2p) = \mathrm{E}[\pi_1] + \left(p - \frac{1}{2}\right)(2p(1-p)), \qquad \text{(S.30)}$$

$$\mathrm{E}[\pi_5] = \mathrm{E}[\pi_6] = p^2(1+6p-10p^2+4p^3) = \mathrm{E}[\pi_3] + \left(p - \frac{1}{2}\right)(2p(1-p))^2. \qquad \text{(S.31)}$$

S.2.3.2 One person having a higher ability than others

Here, we assume an individual who has a higher ability $q(> p)$ than the others, called an *expert*, in the sequential decision-making of individuals with the same ability p discussed so far. The expert is assumed to make a decision at the n-th earliest ($n \geq 3$), and the mean performance is denoted by $\mathrm{E}[\pi_{n,q}]$, as explained in the main text.

First, we assume that the expert knows his/her ability q. The optimal behaviour of

the expert can be summarised according to the difference d_n between $|S_{n-1}|$ and $|T_{n-1}|$ in his/her antecedents as follows:

- In the case of $d_n = 2$, the expert answers s regardless of his/her primary choice as the optimal behaviour if $|S_{n-1}|r^* > |T_{n-1}|r^* + \log[q/(1-q)]$, which is equivalent to $q < e^{2r^*}/\left[1 + e^{2r^*}\right] = p^2/\left[p^2 + (1-p)^2\right] = \pi_{\max}(p)$, by Table 1 in the main text. Otherwise, his/her optimal behaviour is giving his/her primary choice as the answer.

- In the case of $|d_n| = 1$ or $|d_n| = 0$, his/her optimal behaviour is giving his/her primary choice as the answer because q is greater than p and he/she should be a casting-voter even if his/her ability were $p(< q)$ in these cases.

- In the case of $d_n = -2$, by a similar discussion to that in the case of $d_n = 2$, the expert answers t regardless of his/her primary choice as the optimal behaviour if $q < \pi_{\max}(p)$. Otherwise, his/her optimal behaviour is giving his/her primary choice as the answer.

In summary, when $q > \pi_{\max}(p)$, the expert should be a casting voter, and his/her conditional performance and mean performance are q. When $q < \pi_{\max}(p)$, the expert's ability is not so high; therefore, he/she should always give the majority alternative out of the opinions expressed by his/her antecedents if $|d_n| = 2$ and should be a casting voter if $|d_n|$ is 1 or 0; this optimal behaviour is the same as that in the case where the n-th respondent

has the ability p. The mean performance $\mathrm{E}[\pi_{n,q}]$ of the expert when $q < \pi_{max}(p)$ is

$$\begin{aligned}
\mathrm{E}[\pi_{n,q}] &= \sum_{m=0}^{k-2} [2p(1-p)]^m p^2 + [2p(1-p)]^{k-1} q \\
&= \frac{1-(2p(1-p))^{k-1}}{1-(2p(1-p))} p^2 + (2p(1-p))^{k-1} q \\
&= \frac{p^2}{p^2+(1-p)^2} - \left(\frac{p^2}{p^2+(1-p)^2} - q \right) (2p(1-p))^{k-1} \\
&\xrightarrow[k\to\infty]{} \frac{p^2}{p^2+(1-p)^2},
\end{aligned} \tag{S.32}$$

where $n = 2k-1$ or $n = 2k$ $(k = 1, 2, ...)$.

Therefore, the mean performance $\mathrm{E}[\pi_{n,q}]$ of the n-th respondent ($n = 2k-1$ or $n = 2k$) with ability q who knows his/her ability, is summarised as follows:

$$\mathrm{E}[\pi_{n,q}] = \begin{cases} \dfrac{p^2}{p^2+(1-p)^2} - \left(\dfrac{p^2}{p^2+(1-p)^2} - q \right) (2p(1-p))^{k-1} \\ \qquad \xrightarrow[k\to\infty]{} \dfrac{p^2}{p^2+(1-p)^2}, \text{ if } q < \pi_{\max}(q), \\ q, \text{ otherwise.} \end{cases} \tag{S.33}$$

Subsequently, we assume that the expert is not aware of his/her superiority to others in terms of ability and believes that her ability is the same as the others, p. In this case, the expert takes on optimal behaviour for a respondent whose ability is p. Therefore, his/her answer will be the opinion shared by the majority of antecedents when $|d_n| = 2$, and he/she should always give her primary choice as the answer when $|d_n|$ is 1 or 0. Thus,

the mean performance of the expert who believes that his/her ability is the same as that of the others can be written as follows:

$$\begin{aligned}\mathrm{E}[\pi_{n,q}] =& \frac{p^2}{p^2+(1-p)^2} - \left(\frac{p^2}{p^2+(1-p)^2} - q\right)(2p(1-p))^{k-1} \\ &\xrightarrow[k\to\infty]{} \frac{p^2}{p^2+(1-p)^2},\end{aligned} \tag{S.34}$$

where $n = 2k - 1$ or $n = 2k$ $(k = 1, 2, ...)$.

S.2.3.3 Effective size

We evaluated the sequential decision-making of individuals with ability p by its *effective number* of voters, which is defined as follows. Let us denote the mean performance of the simultaneous decision-making of n individuals with the same ability p by $\mathrm{E}[\pi_n^C]$. The effective number n_e of voters in sequential decision-making is the number of voters in which the mean performance of their simultaneous decision-making is equal to the upper bound of the mean performance in their sequential decision-making:

$$\mathrm{E}[\pi_{n_e}^C] = \sum_{m=(n_e+1)/2}^{n_e} \binom{n_e}{m} p^m (1-p)^{n_e-m} = \pi_{\max}(p), \tag{S.35}$$

where n_e is odd, as generally assumed in the literature on collective intelligence [5].

The effective number n_e of voters in the sequential decision-making of individuals with

the same ability is greater than three because

$$\frac{p^2}{p^2+(1-p)^2} - \mathrm{E}[\pi_3^C] = \frac{p^2}{p^2+(1-p)^2} - \left({}_3C_2 p^2(1-p) + p^3\right)$$
$$= \frac{2p^2(1-p)^2(2p-1)}{p^2+(1-p)^2} > 0, \qquad \text{(S.36)}$$

as $p > 0.5$.

Whether or not the effective number is greater than five depends on p because

$$\frac{p^2}{p^2+(1-p)^2} - \mathrm{E}[\pi_5^C] = \frac{p^2}{p^2+(1-p)^2} - \left({}_5C_3 p^3(1-p)^2 + {}_5C_4 p^4(1-p) + p^5\right)$$
$$= \frac{-p^2(1-p)^2(2p-1)(6p^2-6p+1)}{p^2+(1-p)^2}$$
$$= \frac{-p^2(1-p)^2(2p-1)}{p^2+(1-p)^2}\left(p - \frac{3+\sqrt{3}}{6}\right)\left(p - \frac{3-\sqrt{3}}{6}\right). \qquad \text{(S.37)}$$

The effective number is greater than five when $p > (3+\sqrt{3})/6$ and less than five when $0.5 < p < (3+\sqrt{3})/6$.

The effective number is shown to be less than seven, as follows:

$$\frac{p^2}{p^2+(1-p)^2} - \mathrm{E}[\pi_7^C] = \frac{p^2}{p^2+(1-p)^2} - \left({}_7C_4 p^4(1-p)^3 + {}_7C_5 p^5(1-p)^2 + {}_7C_6 p^6(1-p) + p^7\right)$$
$$= \frac{p^2(1-p)^2(2p-1)}{p^2+(1-p)^2}(20p^4 - 40p^3 + 24p^2 - 4p - 1). \qquad \text{(S.38)}$$

Here, $f(p) := 20p^4 - 40p^3 + 24p^2 - 4p - 1$ is negative when $p \in (0.5, 1)$ because

$$\begin{aligned}
f'(p) &= 80p^3 - 120p^2 + 48p - 4 \\
&= 40(2p-1)\left(p - \frac{5+\sqrt{15}}{10}\right)\left(p - \frac{5-\sqrt{15}}{10}\right),
\end{aligned} \tag{S.39}$$

$f(0.5) = -0.75$, $f\left((5+\sqrt{15})/10\right) \sim -1.2$, and $f(1) = -1$. $f(p)$ reaches a local minimum value at $p = (5+\sqrt{15})/10$ and is always negative for $(0.5, 1)$.

Finally, we show that $\mathrm{E}[\pi^C_{n+2}]$ is greater than $\mathrm{E}[\pi^C_n]$.

$$\begin{aligned}
\mathrm{E}[\pi^C_n] &= \sum_{m=(n+1)/2}^{n} {}_nC_m p^m (1-p)^{n-m} \\
&= \sum_{m=(n+1)/2}^{n} {}_nC_m p^m (1-p)^{n-m} (p + (1-p))^2 \\
&= \sum_{m=(n+1)/2}^{n} {}_nC_m \left[p^{m+2}(1-p)^{n-m} + 2p^{m+1}(1-p)^{n-m+1} + p^m(1-p)^{n-m+2}\right] \\
&= \sum_{l=(n+5)/2}^{n+2} {}_nC_{l-2} \left[p^{l}(1-p)^{n-l+2} + 2p^{l-1}(1-p)^{n-l+3} + p^{l-2}(1-p)^{n-l+4}\right],
\end{aligned} \tag{S.40}$$

where $l := m + 2$. Therefore,

$$\begin{aligned}
\mathrm{E}[\pi_n^C] =& {}_nC_{\frac{n+1}{2}} \left[p^{\frac{n+5}{2}}(1-p)^{\frac{n-1}{2}} + 2p^{\frac{n+3}{2}}(1-p)^{\frac{n+1}{2}} + p^{\frac{n+1}{2}}(1-p)^{\frac{n+3}{2}}\right] \\
&+ {}_nC_{\frac{n+3}{2}} \left[p^{\frac{n+7}{2}}(1-p)^{\frac{n-3}{2}} + 2p^{\frac{n+5}{2}}(1-p)^{\frac{n-1}{2}} + p^{\frac{n+3}{2}}(1-p)^{\frac{n+1}{2}}\right] \\
&+ {}_nC_{\frac{n+5}{2}} \left[p^{\frac{n+9}{2}}(1-p)^{\frac{n-5}{2}} + 2p^{\frac{n+7}{2}}(1-p)^{\frac{n-3}{2}} + p^{\frac{n+5}{2}}(1-p)^{\frac{n-1}{2}}\right] \\
&+ \cdots \\
&+ {}_nC_{n-2} \left[p^{n}(1-p)^{2} + 2p^{n-1}(1-p)^{3} + p^{n-2}(1-p)^{4}\right] \\
&+ {}_nC_{n-1} \left[p^{n+1}(1-p) + 2p^{n}(1-p)^{2} + p^{n-1}(1-p)^{3}\right] \\
&+ {}_nC_{n} \left[p^{n+2} + 2p^{n+1}(1-p) + p^{n}(1-p)^{2}\right]. \qquad \text{(S.41)}
\end{aligned}$$

Considering ${}_nC_m = {}_{n-1}C_{m-1} +_{n-1} C_m$ and ${}_nC_m = {}_{n-2}C_{m-2} + 2_{n-2}C_{m-1} + {}_{n-2}C_m$,

$$
\begin{aligned}
\mathrm{E}[\pi_n^C] =& {}_nC_{\frac{n+1}{2}}\ p^{\frac{n+1}{2}}(1-p)^{\frac{n+3}{2}} + \left(2_nC_{\frac{n+1}{2}} + {}_nC_{\frac{n+3}{2}}\right) p^{\frac{n+3}{2}}(1-p)^{\frac{n+1}{2}} \\
&+ \sum_{i=\frac{n+5}{2}}^{n} {}_{n+2}C_i\ p^i(1-p)^{n+2-i} + (n+2)p^{n+1}(1-p) + p^{n+2} \\
<& {}_nC_{\frac{n+1}{2}}\ p^{\frac{n+3}{2}}(1-p)^{\frac{n+1}{2}} + \left(2_nC_{\frac{n+1}{2}} + {}_nC_{\frac{n+3}{2}}\right) p^{\frac{n+3}{2}}(1-p)^{\frac{n+1}{2}} \\
&+ \sum_{i=\frac{n+5}{2}}^{n+2} {}_{n+2}C_i\ p^i(1-p)^{n+2-i}, \\
=& \left({}_nC_{\frac{n+1}{2}} + 2_nC_{\frac{n+1}{2}} + {}_nC_{\frac{n+3}{2}}\right) p^{\frac{n+3}{2}}(1-p)^{\frac{n+1}{2}} + \sum_{i=\frac{n+5}{2}}^{n+2} {}_{n+2}C_i\ p^i(1-p)^{n+2-i} \\
=& {}_{n+2}C_{\frac{n+3}{2}} p^{\frac{n+3}{2}}(1-p)^{\frac{n+1}{2}} + \sum_{i=\frac{n+5}{2}}^{n+2} {}_{n+2}C_i\ p^i(1-p)^{n+2-i} \\
=& \sum_{i=\frac{n+3}{2}}^{n+2} {}_{n+2}C_i\ p^i(1-p)^{n+2-i} \\
=& \mathrm{E}[\pi_{n+2}^C],
\end{aligned}
$$

where the inequality above holds because $p^{\frac{n+1}{2}}(1-p)^{\frac{n+3}{2}} < p^{\frac{n+3}{2}}(1-p)^{\frac{n+1}{2}}$.

To sum up, the effective number of voters in sequential decision-making is between 3 and 5 when $0.5 < p < (3+\sqrt{3})/6$, and is between 5 and 7 when $(3+\sqrt{3})/6 < p < 1$. Here, $(3+\sqrt{3})/6 \sim 0.79$.

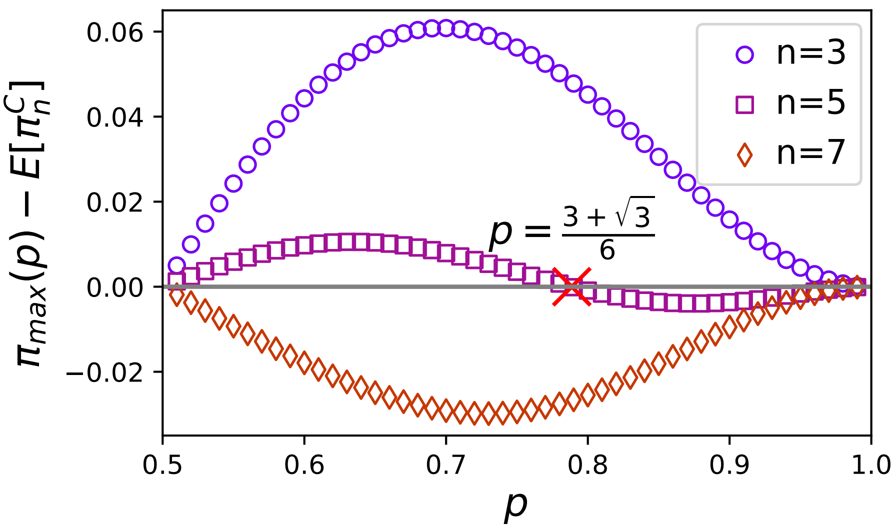

Figure S.4: $\pi_{max}(p) - \mathrm{E}[\pi_n^C]$ versus p for $n = 3, 5, 7$.

References

[1] Nitzan S. Collective preference and choice. Cambridge University Press; 2009.

[2] Shapley L, Grofman B. Optimizing group judgmental accuracy in the presence of interdependencies. Public Choice. 1984;43(3):329–343.

[3] Nitzan S, Paroush J. Optimal decision rules in uncertain dichotomous choice situations. International Economic Review. 1982:289–297.

[4] Marshall JA, Brown G, Radford AN. Individual confidence-weighting and group decision-making. Trends in ecology & evolution. 2017;32(9):636–645.

[5] Ladha KK. The Condorcet jury theorem, free speech, and correlated votes. American Journal of Political Science. 1992:617–634.
